# Uncovering the Role of Land Use Intensity in Shaping Forest and Grassland‐Specific Soil Fungal Communities

**DOI:** 10.1111/1462-2920.70170

**Published:** 2025-08-22

**Authors:** Rosario Iacono, François Buscot, Spaska Forteva, Ingo Schöning, Marion Schrumpf, Emily Solly, Stephan Wöllauer, Kezia Goldmann

**Affiliations:** ^1^ Department of Soil Ecology Helmholtz‐Centre for Environmental Research–UFZ Halle (Saale) Germany; ^2^ German Centre for Integrative Biodiversity Research (iDiv) Halle‐Jena‐Leipzig Leipzig Germany; ^3^ Faculty of Geography Philipps‐University of Marburg Marburg Germany; ^4^ Department for Biogeochemical Processes Max‐Planck‐Institute for Biogeochemistry Jena Germany; ^5^ Department of Computational Hydrosystems Helmholtz Centre for Environmental Research – UFZ Leipzig Germany; ^6^ Faculty of Resource Management HAWK University of Applied Sciences and Arts Göttingen Germany

## Abstract

Soil fungal communities are shaped by land use intensity (LUI) and environmental conditions, but their combined effects remain unclear. Using data from 300 forest and grassland plots across Germany from 2021, we analysed fungal taxa relative abundance and associations with environmental variables. Soil conditions, soil fungal diversity, and community composition were linked to ecosystem variables and differed significantly across LUI levels. Forests showed greater variation in soil conditions across LUI levels; grasslands displayed the most pronounced differences in fungal diversity. In forest ecosystems, taxa belonging to the classes *Leotiomycetes* and *Sordariomycetes* (all *Pezizomycotina*) were indicators under both high and low LUI levels (*R* > 0.55, *p* < 0.005). High LUI in forests was characterised by a higher ratio between *Basidiomycota* and *Ascomycota*. In grasslands, high LUI levels were associated with more indicator taxa from *Mortierellomycota* and fewer from *Glomeromycota* compared to low LUI levels (*R* > 0.6, *p* < 0.005). This is the first comprehensive study addressing differences in soil fungal communities between grasslands and forests and across management intensities in Europe. Our findings suggest differential response of the two ecosystems to changes in LUI, with forests having more resilient traits in terms of soil fungal community richness and composition, while grassland fungi appear more sensitive to management practices.

## Graphical abstract

We analysed soil fungal diversity in 300 forest and grassland plots across Germany, assessing land use intensity (LUI) effects. Forests showed stronger soil condition variation, while grasslands exhibited larger diversity shifts. We identified key fungal indicator species and trophic guilds linked to LUI, revealing their ecosystem roles.

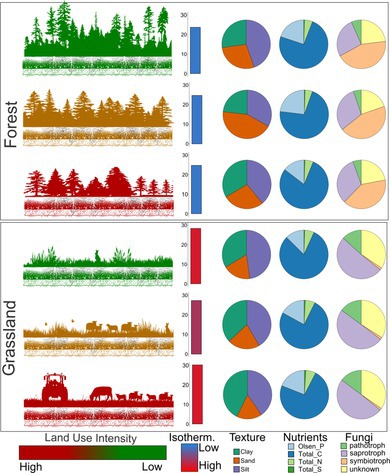

## 1 Introduction

Land use intensity (LUI) is a key driver of biodiversity loss in terrestrial ecosystems (Felipe‐Lucia et al. 2020). However, its specific impact on soil fungi across Europe's major biomes—forests and grasslands—remains poorly understood (Spurgeon et al. 2013; Castaño et al. 2018; Labouyrie et al. 2023). Forests and grasslands dominate the European landscape (Eurostat 2018), and their management varies from low‐intensity, semi‐natural systems to highly intensive, production‐focused regimes (Mason et al. 2022). These management gradients create a mosaic of ecological conditions that shape both above‐ and belowground biodiversity (Guasconi et al. 2023).

Soil fungi are central to ecosystem functioning as they drive essential processes such as nutrient cycling, carbon sequestration, and other determinants of soil health (Cardinale et al. 2012; Hooper et al. 2012). Fungi form highly diverse and specialised communities that differ markedly between forests and grasslands. In temperate forests, ectomycorrhizal fungi (EMF) dominate and form symbiotic relationships with trees, which facilitate nutrient uptake and enhance plant health (Tedersoo et al. 2014; Labouyrie et al. 2023). In contrast, grassland plants mainly associate with arbuscular mycorrhizal fungi (AMF) that play a key role in supporting herbaceous communities (Smith and Read 2008; Gao and Guo 2010). Beyond mycorrhizal associations, fungi also act as saprotrophs, decomposing organic matter, and as mutualists or pathogens engaged in complex interactions with other soil organisms (McLaughlin and Spatafora 2014; Purahong et al. 2018).

The diversity and function of soil fungi are highly modulated by both land use and environmental changes (Mukhtar et al. 2021; Zhang et al. 2024; Zhou et al. 2024). Numerous studies have shown that intensified land use, for example, conversion of forests or grasslands to croplands, typically reduces soil microbial and fungal biomass and diversity, often more pronounced for fungi than for bacteria (Siles et al. 2023; Beugnon et al. 2025). For example, Siles et al. (2023) found significantly lower fungal biomass in cropland soils across Europe compared to more natural land uses, which they attributed to a higher susceptibility of fungi to intensive management. Conversely, extensive, low‐intensity land use supports higher soil microbial activity, biomass, and fungal‐to‐bacterial ratios (Sünnemann et al. 2021; Beugnon et al. 2025). These patterns highlight the vulnerability of soil fungi to anthropogenic pressures and underscore their importance as indicators of ecosystem health.

Climatic factors, particularly precipitation and temperature, shape fungal community composition and often modulate the direction and magnitude of land use effects (Mukhtar et al. 2021; Siles et al. 2023; Djemiel et al. 2024). Soil properties such as pH, organic matter content and nutrient availability further influence fungal community composition and functioning (Zhang et al. 2016; Luo et al. 2021; Liu et al. 2025). Importantly, the interplay between climatic and edaphic factors can either buffer or amplify the impacts of LUI, leading to context‐dependent responses across biomes and regions (Zhou et al. 2024).

The fungal metabolic versatility underpins ecosystem diversity, productivity, and resilience (Brundrett 2002; Van Der Heijden et al. 2008; Fellbaum et al. 2012; Gmach et al. 2020; Buscot 2023). The resilience of fungal communities to land‐use changes is thus a key predictor of long‐term ecosystem stability. Current research identifies three key indicators of fungal community resilience: (1) Functional redundancy, that is, the maintenance of ecosystem processes despite species loss, measurable through metagenomic potential or enzyme activity assays (De Vries et al. 2018); (2) recovery trajectories, inferred from compositional shifts after disturbance cessation, which is reflected by temporal β‐diversity metrics (Allison and Martiny 2008); and (3) stress‐tolerant taxa persistence, identifiable via abundance thresholds of documented stress responders (e.g., certain *Mortierellaceae*; Banerjee et al. 2019). While longitudinal data remain ideal, such indicators can also be approximated in large‐scale cross‐sectional studies by comparing functional guild diversity, community similarity decay rates, and the prevalence of stress‐response traits (Peay et al. 2012; Hannula and Morriën 2022). Disentangling the effects of LUI from those of environmental variables is essential for understanding and managing biodiversity loss and ecosystem service degradation. Land use and environmental factors are deeply intertwined: LUI alters local environmental conditions (e.g., soil properties or microclimate), while climate independently shapes species distributions and ecological processes (Smith et al. 2019; Redlich et al. 2022; Zheng et al. 2022). These interactions create feedback loops that complicate causal inference and attribution in ecological studies. Notably, the independent contributions of LUI versus land use type (e.g., forest vs. cropland) remain poorly quantified, despite evidence that intensity changes often dominate ecosystem service trade‐offs (Zheng et al. 2022).

Despite the recognised importance of these drivers for developing targeted conservation policies and sustainable land management strategies, few studies have systematically disentangled the independent and the combined effects of LUI and environmental variables on soil fungi at large spatial scales (Maloney et al. 2020; Redlich et al. 2022; Zheng et al. 2022). This knowledge gap limits our ability to predict and mitigate biodiversity loss and ecosystem service degradation under ongoing land use and climate change.

In this study, we investigated how soil fungal diversity and community composition in forests and grasslands is affected by LUI and environmental variation. We analysed soil fungal diversity and composition in 150 forest and 150 grassland plots across Germany, using environmental and land use data from the Biodiversity Exploratories (Fischer et al. 2010). Our objectives were to (a) identify fungal phyla and trophic groups most affected by LUI in both ecosystem types; (b) assess the mediating role of soil climatic and physical conditions shaping fungal communities; (c) determine fungal taxa that serve as indicators of land use in both forests and grasslands; and (d) compare the magnitude of LUI‐driven changes between these two ecosystem types. We hypothesised that: (i) environmental gradients within each biome further structure fungal communities, potentially overriding the biome‐level patterns (Tedersoo et al. 2014; Bahram and Netherway 2022); (ii) increasing LUI leads to ecosystem‐specific shifts in fungal diversity and composition, which potentially promote pathogenic fungi in forests and alter saprotrophic communities in grasslands (Labouyrie et al. 2023); and (iii) bioclimatic variables, particularly soil temperature and moisture, create stable microenvironments that significantly influence fungal community structure.

By disentangling the effects of LUI and environmental variation, our work aims to advance understanding of soil fungal ecology and inform strategies for biodiversity conservation and sustainable land management in European landscapes.

## 2 Material and Methods

### 2.1 Study Sites and Sampling

Our study utilised a comprehensive dataset generated within the framework of the German Biodiversity Exploratories, which comprise 300 experimental plots (EPs) distributed across Germany and located in three geographically separated regions: Schwäbische Alb (South‐West), Hainich‐Dün (Central), and Schorfheide‐Chorin (North‐East) (Fischer et al. 2010). These regions differ markedly in their topo‐geographical and soil chemical and physical properties (Fischer et al. 2010; Bramble et al. 2024). Each region contains 50 grassland and 50 forest EPs, representing a range of land use intensities. Grassland EPs measure 50 m × 50 m, while forest EPs are 100 m × 100 m.

LUI was assessed separately for grassland and forests. In grasslands, LUI indices were calculated according to Blüthgen et al. (2012), based on data provided by the land owners regarding mowing, grazing and fertilisation (Vogt et al. 2019), using the LUI calculation tool (Ostrowski et al. 2020) available in BExIS (http://doi.org/10.17616/R32P9Q). For forests, LUI was estimated using the silvicultural management index as defined by Schall and Ammer (2013). Each EP was categorised into one of three LUI levels (low, medium, high) based on its individual index value, using thresholds set at the 33rd and 66th percentiles. We chose this three‐level categorisation to capture potential effects of non‐linearity of management. This resolution allowed us to detect patterns and ecological responses that might be overlooked with a simple binary classification, providing a more nuanced understanding of how varying degrees of land use impact soil fungi. For the differential expression analysis only, the EPs at the highest and lowest LUI levels were considered, focusing on the most contrasting management conditions.

Soil sampling was performed almost simultaneously across all 300 EPs in early May 2021. This timing was selected to optimise the detection of land management effects on soil microbial communities, as sampling later in the season could introduce confounding effects such as drought (Nannipieri et al. 2019). At each EP, 14 soil cores (5 cm diameter and 10 cm depth after removal of turf or organic litter) were collected along two orthogonal transects, 20 m in grasslands and 40 m in forests. The 14 soil cores were pooled to one composite soil sample, to focus on between‐plot rather than within‐plot variation.

To account for regional differences in soil texture, pooled samples were sieved to < 2 mm in Schorfheide‐Chorin and to < 4 mm in Hainich‐Dün and Schwäbische Alb, to effectively remove stones and roots. From each pooled sample, 500 g was dried at 40°C for determination of physico‐chemical soil properties, while 15 g were stored at ‐20°C for subsequent molecular analysis of soil fungi. For molecular analyses, samples were collected and immediately transported in cooling bags to maintain a constant low temperature. Upon arrival at the field laboratory, samples were sieved and frozen at ‐20°C. All samples were stored at ‐20°C within 5 h of collection according to our established protocol.

### 2.2 Determination of Soil Physico‐Chemical Properties and Bioclimatic Variables

The physical characteristics of the soil were defined by measuring soil texture, pH, carbon, nitrogen, sulphur and plant‐available phosphorus contents on air‐dried samples.

The texture analysis included three steps: (i) destruction of soil organic matter with hydrogen peroxide, (ii) dispersion of soil aggregates into discrete units, and (iii) separation of soil particle sizes by sieving and sedimentation according to DIN‐ISO 11277 (Schöning et al. 2021).

For soil pH analysis, 10 g of air‐dried soil was mixed with 25 mL 0.01 M CaCl_2_ solution and shaken for 2 h. Subsequently, the pH of each soil suspension was measured twice using a glass electrode (Schöning 2024).

Total C, N and S concentrations were determined by dry combustion in an elemental analyser (VarioMax) at a temperature of 1100°C. The evolving CO_2_, N_2_, SO_2_ were determined with a Thermal Conductivity Detector (TCD) after being calibrated with glutamic acid. Inorganic carbon (IC) was determined after removing organic carbon (OC) by ignition at 450°C for 16 h using the same elemental analyser. OC was calculated as the difference between total carbon (TC) and IC. The C:N (CN) and C:S (CS) ratios were calculated as OC mass to total nitrogen (TN) and total sulphur (TS) mass, respectively.

For analysing plant‐available phosphorus (OP), soil samples were shaken with 0.5 M NaHCO_3_ (pH of 8.5). Phosphate was quantified photometrically in filtered extracts as a blue coloured molybdate complex (*λ* = 880 nm; Schöning 2025) using a flow injection system (Lachat Quickchem QC85S5).

Each EP had a climate station providing climate data (Wöllauer et al. 2021; Hänsel et al. 2024). This study focused on temperature and moisture in the first 10 cm of soil from 2017 to 2021 (Table S1), which were averaged across years.

### 2.3 DNA Extraction and ITS Amplicon Library Preparation

Genomic DNA was extracted from each pooled soil sample using the Power Soil DNA Isolation Kit (Qiagen) following the manufacturer's protocol. DNA concentrations were quantified with a NanoDrop UV–Vis spectrophotometer (Peqlab), and all extracts were equally diluted prior to PCR.

The fungal internal transcribed spacer region 2 (ITS2) was amplified using the primer set fITS7 and ITS4 (Gardes and Bruns 1993; Ihrmark et al. 2012). The 18 μL PCR reaction mixture consisted of 10 μL 2 × KAPA HiFi HotStart ReadyMix DNA polymerase (ROCHE), 0.4 μL of each primer, and 2 μL of DNA template. PCR conditions were 95°C for 3 min, followed by 30 cycles of 98°C for 20 s, 56°C for 20 s and 72°C for 20 s, with a final extension of 72°C for 5 min. Each sample was amplified twice, accompanied by one negative control per PCR plate. Amplifications were verified by agarose gel electrophoresis with ethidium bromide staining on a 1.5% agarose gel. The amplicon duplicates were pooled and purified with AMPure XP beads (Beckman Coulter). These purified products served as templates in a subsequent PCR, introducing the Illumina Nextera XT indices and sequencing adaptors according to the manufacturer's instructions. The PCR conditions were as follows: initial denaturation at 95°C for 3 min, eight cycles of denaturation at 98°C for 30 s, annealing at 55°C for 30 s, followed by elongation at 72°C for 30 s, and a final extension at 72°C for 5 min. Resulting PCR products were purified again with AMPure beads. The amplicon libraries were quantified using PicoGreen assays (Molecular Probes) and pooled equimolarly. Fragment sizes and the library quality were checked using an Agilent 2100 Bioanalyzer (Agilent Technologies). The final pool was used for paired‐end sequencing of 2 × 300 bp with a MiSeq Reagent kit v3 on an Illumina MiSeq platform at the Department of Soil Ecology of the Helmholtz‐Centre for Environmental Research—UFZ in Halle (Saale), Germany. Raw sequences were deposited in the National Center for Biotechnology Information (NCBI) Sequence Read Archive (SRA) under study accession number PRJNA1213839.

### 2.4 Bioinformatic Processing and Ecological Grouping

Raw forward and reverse ITS2 reads were demultiplexed by the Illumina reporter software v2.5.1.3, providing fastq files with the Illumina adaptors, indices and sequencing primers removed. Further downstream processing used the DADA2 (Callahan et al. 2016) based pipeline dadasnake v0.11 (Weißbecker et al. 2020). Amplification primers were removed using cutadapt v1.18 (Martin 2011), setting the minimum read length of 70 bp and Phred score of 20. Sequences were merged with at least 20 bp overlap, and chimeric sequences were removed. Amplicon sequence variants (ASVs; Callahan et al. 2017) were generated and then clustered at 97% sequence similarity using VSEARCH v2.22 (Rognes et al. 2016) to account for intraspecific ITS variability (Estensmo et al. 2021). Hence, we refer to operational taxonomic units (OTUs) as a fungal species equivalent. The consensus sequences of each OTU were taxonomically assigned using the MOTHUR implementation of the Bayesian classifier (Schloss et al. 2009) and the database UNITE, version 9.0 (Abarenkov et al. 2024) within dadasnake (Weißbecker et al. 2020). All OTUs without a phylum annotation or not of fungal origin according to ITSx v1.1.3 (Bengtsson et al. 2019) were removed. FungalTraits v1.2 (Põlme et al. 2020) was used to parse fungal taxonomy and determine fungal lifestyles.

### 2.5 Data Analyses

The statistical analyses of the data were performed in R version 4.3.0 (R Core Team 2023). Bioclimatic variables were calculated from climate station data (see 2.2) using a modified version of function biovar in package Dismo (Hijmans et al. 2023), following Nix (1986) and Hijmans et al. (2005), with a detailed list of bioclimatic variables in Table 1.

**TABLE 1:** List of soil bioclimatic variables calculated from soil temperature and moisture values in the first 10 cm of soil using the BIOCLIM approach as described by Booth (2018).

	Bioclimatic variable	Description
Soil temperature	bio 1	Soil annual mean temperature [°C]
	bio 2	Mean diurnal range (Mean (period max‐min)) [°C]
	bio 3	Isothermality (bio2/bio7) [°C]
	bio 4	Temperature seasonality (standard deviation) [°C]
	bio 5	Max temperature of warmest period [°C]
	bio 6	Min temperature of coldest period [°C]
	bio 7	Temperature annual range (bio5‐bio6) [°C]
	bio 8	Mean soil temperature of wettest quarter [°C]
	bio 9	Mean soil temperature of driest quarter [°C]
	bio 10	Mean temperature of warmest quarter [°C]
	bio 11	Mean temperature of coldest quarter [°C]
Soil moisture	bio 12	Annual average moisture in soil [%]
	bio 13	Soil moisture in the of wettest period [%]
	bio 14	Soil moisture of driest period [%]
	bio 15	Moisture annual range [%]
	bio 16	Soil moisture seasonality (coefficient of variation) [%]
	bio 17	Soil moisture of wettest quarter [%]
	bio 18	Soil moisture of driest quarter [%]
	bio 19	Soil moisture of warmest quarter [%]
	bio 20	Soil moisture of coldest quarter [%]

To account for differences in sampling depth, fungal abundance data were rarefied to an even depth of 42,138 sequences per sample using the rrarefy() function in the vegan package (Oksanen et al. 2022). Sequence abundances were then transformed to relative abundances prior to subsequent statistical analyses.

Fungal community diversity was quantified using Hill numbers, N1 representing the exponential Shannon entropy, and N2 representing the inverse Simpson index. Hill numbers were selected for their ecological interpretability, as they express diversity in units of “effective species” facilitating direct comparisons across ecosystems (Hill 1973; Gotelli and Chao 2013). These diversity metrics were calculated using the package vegan (Oksanen et al. 2022). Details of the diversity indices are presented in Table S2.

Linear mixed models were employed to assess the effect of ecosystem type and LUI on environmental and soil variables, as well as fungal diversity indices. Elevation and Exploratory were included as random effects to account for environmental heterogeneity and improve estimation of fixed effects. Model significance was evaluated using analysis of variance (ANOVA), with significance set to *p* < 0.01. Significant interactions were prioritised over main effects. For LUI, only the highest and lowest intensity levels were compared. Significant effects were further investigated using estimated marginal means (EMM, emmeans package, Lenth 2023) with *p* values adjusted via multivariate *t* distribution (mvt) and Satterthwaite method.

Relationships between bioclimatic variables and biodiversity indices, accounting for the Exploratory, were tested by calculating Spearman's rank correlation coefficient (Bonferroni corrected) on residuals of the mixed effect models with Exploratory as a random effect. The Spearman correlation was chosen for its robustness to non‐normality and non‐monotonic relationships.

Differences in fungal OTU relative abundance between ecosystem types and LUI extremes were tested, and fungal β‐diversity was assessed using the Sörensen Baselga index (Baselga 2010) via beta.div.comp in the adespatial package (Dray et al. 2023). Associations between fungal relative abundances and environmental variables were examined using distance‐based redundancy analysis (db‐RDA) (Legendre and Legendre 2012) with forward stepwise selection by permutation (Blanchet et al. 2008). Further details are provided in the supplemental method description.

Fungal indicator species were identified with the INDVAL index (multipatt function in the indicspecies package; Dufrêne and Legendre 1997; Cáceres and Legendre 2009), with *p* values corrected for false discovery rate (FDR; Benjamini‐Hochberg procedure, Benjamini and Hochberg 1995) due to high sample and variable numbers. More details on the indicator species analysis are in the supplemental method description.

## 3 Results

### 3.1 Characterisation of the Ecosystem‐Specific Soil Environment

**FIGURE 1:**
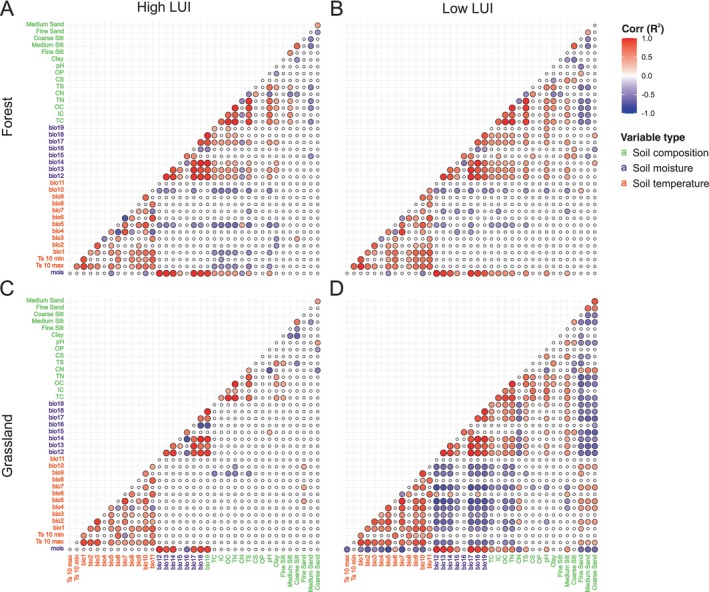
Correlation between environmental variables in four ecosystem/land use intensity combinations: (A) forest, high land use intensity, (B) forest, low land use intensity, (C) grassland, high land use intensity, and (D) grassland, low land use intensity. Correlations were calculated using Pearson correlation on mixed‐effects model residuals, with Bonferroni correction applied for multiple testing. Only significant correlations (adj. *p* < 0.05) are shown. Colour scale shows the correlation coefficient (*R*) from 1 (red) to −1 (violet). Ecosystem/land use intensity combinations displayed distinct correlation strength (*R*) and significance levels (adj. *p*).

Bioclimatic and geochemical properties in the topsoil had consistently higher values in grasslands than in forests (Table S3). LUI levels induced contrasting effects on water content and inorganic carbon between the two ecosystems (Table S4). The correlation between these variables also varied across LUI levels and ecosystems (Figure 1).

Analyses of soil parameters across ecosystems and LUI levels revealed key differences in variables correlations, especially between forests (Figure 1A,B) and grasslands (Figure 1C,D). Soil pH was a major differentiating factor, showing a strong positive association with soil nutrient content and moisture‐related bioclimatic variables across ecosystems and LUI levels (Figure S1). In forests, at both high and low LUI, pH clustered strongly with soil carbon, nitrogen, and sulphate contents (Figure 1A,B). In grasslands, these correlations were generally absent, except for a positive association between pH and C/N ratio under high LUI (Figure 1C), and between pH and soil carbon content under low LUI (Figure 1D). In grasslands under low LUI, bioclimatic variables related to soil temperature (bio1–bio11) exhibit negative correlations with those related to soil moisture (bio12–bio21), a pattern not observed under high LUI levels.

### 3.2 Fungal Diversity

Forests had a lower relative abundance of fungal taxa than grasslands (Table S6). The number of differentially abundant genera in response to LUI was similar between ecosystems (19 in grasslands vs. 16 in forests; Tables S10 and S11), indicating that LUI affects fungal communities in both systems to a comparable extent.

**FIGURE 2:**
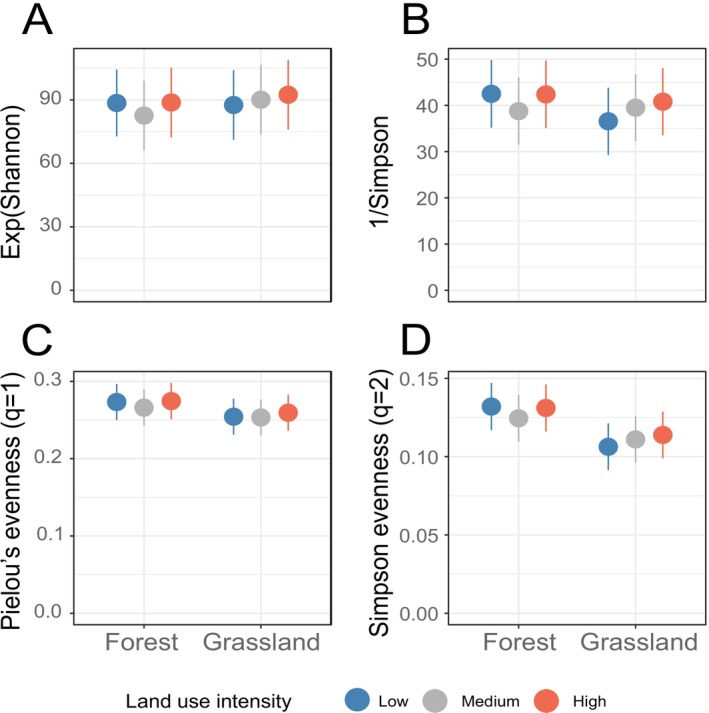
Fungal community diversity response to land‐use intensity across forests and grasslands. (A, B) Hill numbers (N1, N2) showing effective species counts; higher values mean greater diversity. (C, D) Evenness metrics (E1, E2) reflecting abundance distributions, with values near 1 indicate even communities and near 0 suggest dominance of certain taxa. Error bars are 95% confidence intervals from mixed‐model estimated marginal means (emmeans).

**FIGURE 3:**
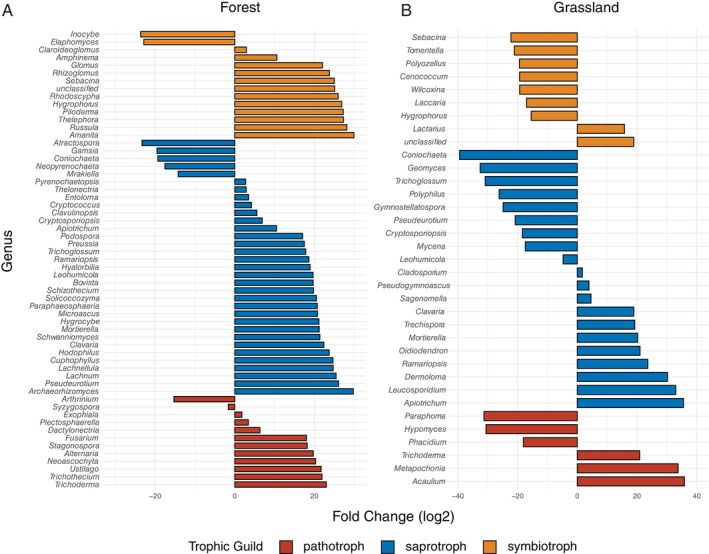
Differentially abundant fungal genera at low or high land use intensity plots in (A) forests and (B) grassland.

**FIGURE 4:**
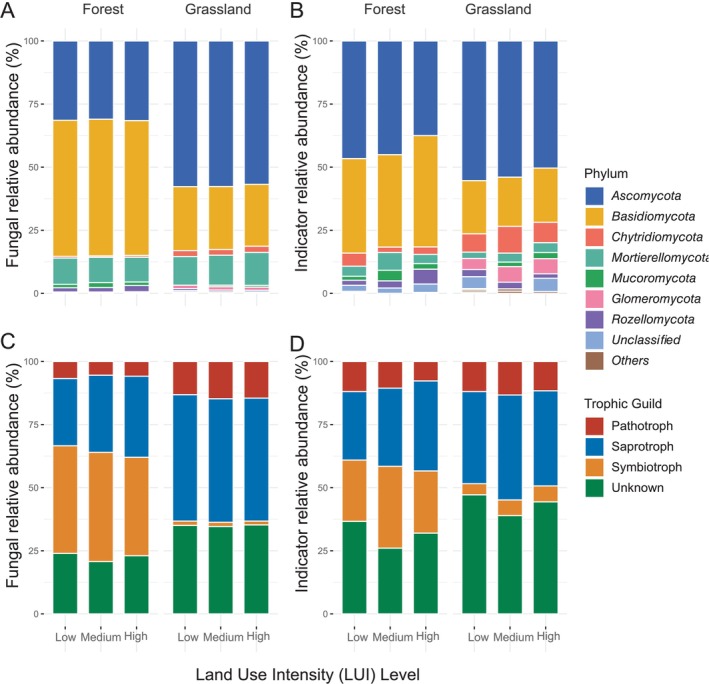
Relative abundance of all soil fungi and indicator species, respectively, in forests and grasslands across different land use intensities grouped by (A, B) fungal phylum, and (C, D) trophic guild. Relative abundances were standardised across conditions.

Analysis of Hill numbers and evenness metrics (Figure 2) revealed distinct patterns between ecosystems under varying LUI. In grasslands, both fungal diversity and evenness tended to increase with higher LUI (low: N1 = 87.593 ± 4.663; medium: 90.114 ± 4.657; high: 92.45 ± 4.667; *p* > 0.05). In forests, high LUI was associated with a higher abundance of certain fungal genera, including known plant pathogens such as *Fusarium* (Figure 3, Table S10). In grasslands, however, while a comparable number of genera exhibited differential abundance between high and low LUI, no consistent trend in their functional roles (e.g., pathogens, saprotrophs) was evident (Table S11). Notably, fungal richness in forests remained stable across LUI levels, whereas grasslands subjected to high LUI harboured significantly more fungal taxa than those under medium or low intensity (Table S6). Both ecosystems were dominated by *Ascomycota*, *Basidiomycota*, and to a lesser extent *Mortierellomycota*, with a higher *Asco*‐ to *Basidiomycota* ratio in grasslands compared to forests (Figure 4A). Similarly, forests were characterised by a lower abundance of pathotrophs and saprotrophs, but more symbionts compared to grasslands (Figure 4C). LUI had little impact on the overall fungal taxonomy and guilds, though the ratio of saprotroph to symbiotrophs was lower at high LUI, and in grasslands, the ratio of pathotrophs to saprotrophs was higher at the high LUI.

The indicator species analyses on fungal OTU level complemented the previous findings, as they showed slightly more fungal indicators in forests than in grasslands (26 in forest vs. 24 in grasslands; Table S12). *Ascomycota* and *Basidiomycota* dominated indicator taxa under all conditions (Figure 4B). However, plots with higher LUI were characterised by a lower ratio of indicator species belonging to *Ascomycota* to those from *Basidiomycota* compared to low LUI. Most of the fungal indicators in grassland were saprotrophs or of unknown lifestyle. In forest, high LUI increased the ratio of pathogens to saprotroph, while in grassland, it increased the ratio of saprotroph to symbiotic and the number of species with unknown lifestyle (Figure 4D).

Fungal β‐diversity across forest and grassland accounted for 43.04% of the total fungal diversity. Of this, 99.08% was due to fungal species replacement, while 0.92% was due to nestedness (Table S9).

### 3.3 Correlations Between Environmental Variables and Fungal Diversity

**FIGURE 5:**
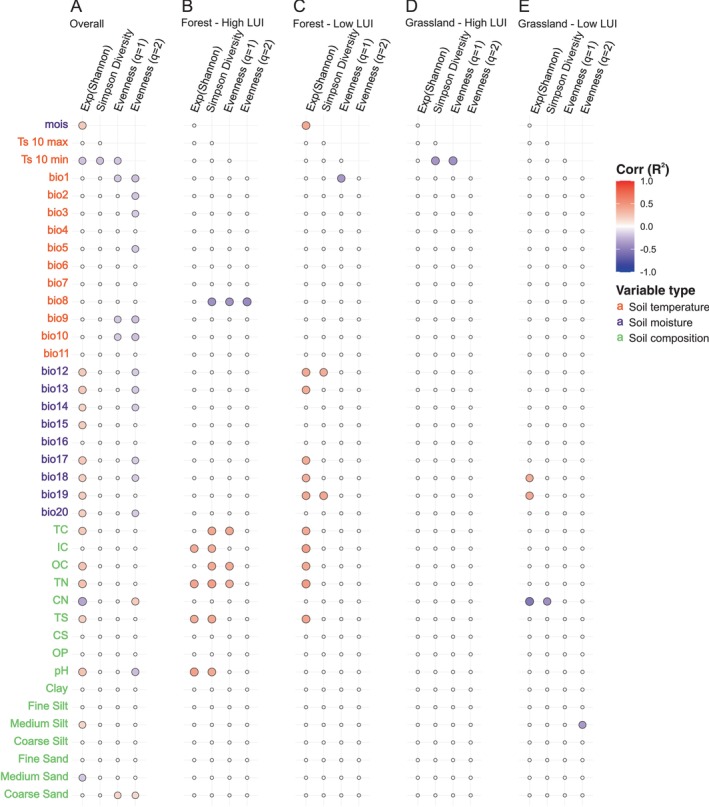
Correlation between environmental variables and fungal diversity indices. Correlations were calculated using Pearson correlation with Bonferroni correction on residuals: (A) all data, (B) forest, high land use intensity (LUI), (C) forest, low LUI, (D) grassland, high LUI, (E) grassland, low LUI. Only significant correlations (adj. *p* < 0.05) are shown. Colour scale indicates correlation coefficient (*R* ^2^) from 1 (blue) to −1 (brown).

Overall, fungal alpha diversity showed a weak positive correlation with the soil properties TC, OC and IC, TN and TS (Figure 5A). These relationships were detected in forests but not in grasslands. However, forests with high LUI had fewer significant correlations between fungal diversity and environmental variables than forests with low LUI. Notably, in forests with high LUI, correlations between fungal richness and TS were absent (Figure 5B,C). For grasslands, independent of LUI level, no correlation between fungal diversity and any environmental factor could be found (Figure 5D,E). Correlation analysis between bioclimatic variables and fungal diversity revealed significant relationships exclusively within forest ecosystems under low LUI. Specifically, fungal diversity was positively correlated with average annual soil moisture (bio12) and soil moisture during the wettest quarter (bio13), while exhibiting a negative correlation with mean soil temperature of the warmest quarter (bio10) (Figure 5C,D).

### 3.4 Effect of Environment on Fungal Communities

**FIGURE 6:**
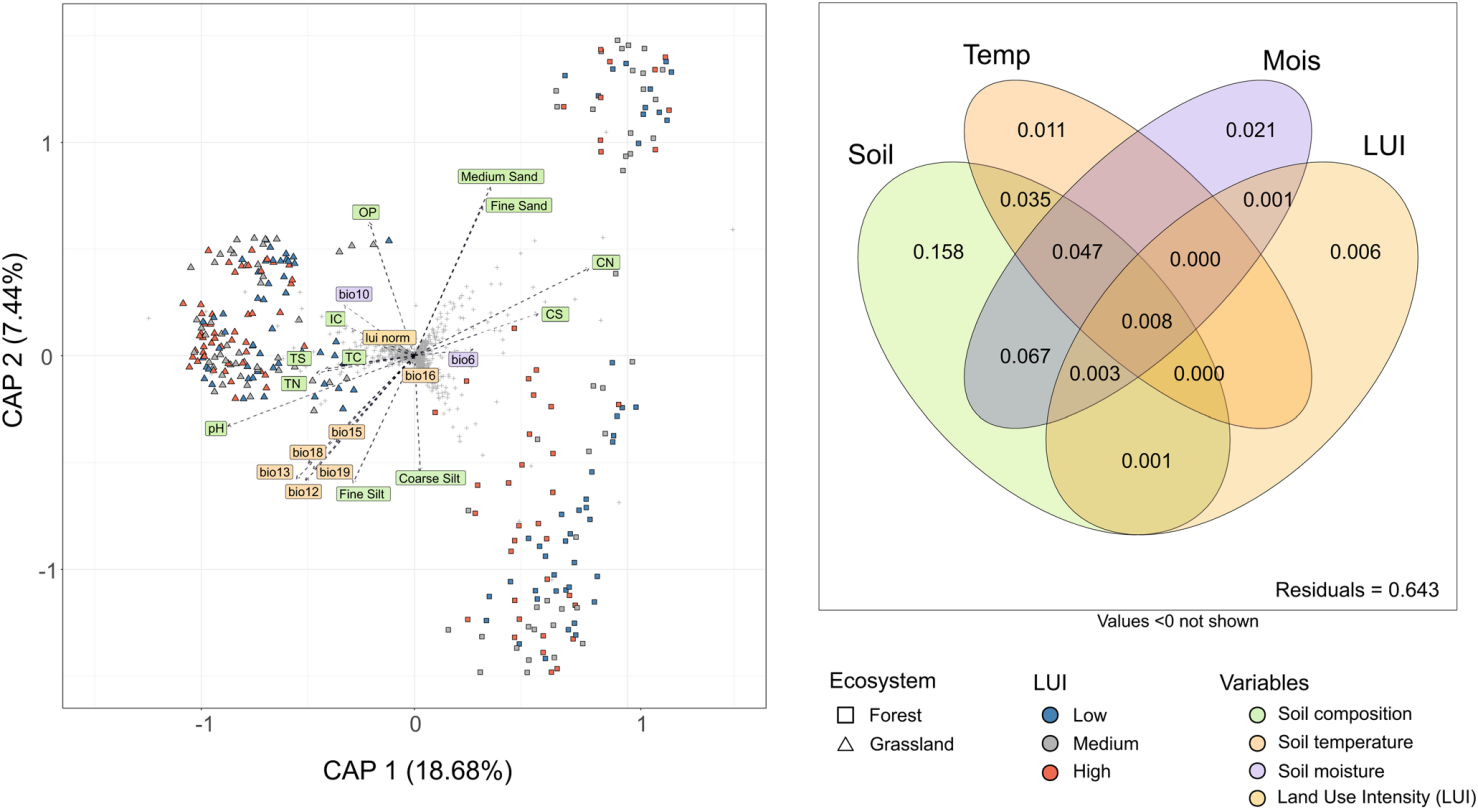
Plot of the variance distribution of the distance‐based redundancy analysis (db‐RDA) between fungal species distribution within the Biodiversity Exploratories in 2021 and environmental conditions from 2017 to 2020 in the same locations. Values indicate adjusted *R* ^2^ values.

Variance partitioning (Figure 6) revealed that the fungal communities were primarily structured by the physicochemical soil properties (adj *R* ^2^ = 0.319), followed by soil moisture (adj *R* ^2^ = 0.145) and soil temperature (adj *R* ^2^ = 0.10), while LUI had only a small contribution (adj *R* ^2^ = 0.02). The basis for these calculations, a db‐RDA model on the full rank distance‐based matrix of fungal relative abundance with the selected set of environmental variables, was highly significant (ANOVA like test, *p* < 0.001, Figure S2A). The first two axes explained 25.89% of the relationship between fungal community and environmental variables, clearly separating fungal assemblages from forests and grasslands. Key variables driving these differences included soil pH, soil moisture, the contents of phosphorus, clay and sand, as well as C/N and C/S ratio.

Independent db‐RDA for each ecosystem type revealed that in forests, the first two axes encapsulated 24.47% of the total variance (Figure S2B); in grasslands, they explained only 23.84% of the total variance (Figure S2C).

## 4 Discussion

While research has traditionally focused on aboveground vegetation, understanding differences in belowground communities between forests and grasslands requires examining environmental soil conditions. By introducing bioclimatic variables tailored to soil environments, our study provides new insights into how soil fungal diversity in German forests and grasslands responds to LUI and local conditions. These variables capture the climatic variability experienced by soil communities, enabling an assessment of fungal responses to environmental change.

Although seasonal variation within individual plots during the year is important and has been investigated previously (Goldmann et al. 2020), the present work focused on the effects of ecosystem type and LUI levels on fungal community in relation to soil conditions.

### 4.1 Drivers of Fungal Community Differences Between Forest and Grassland

While bioclimatic variables are well established in species distribution modelling (Nix 1986; Hijmans and Elith 2013), our study applies them to soil environments, providing a novel perspective on micro‐environmental factors shaping soil communities. The use of bioclimatic variables allows to account for seasonal variations within soils at a single time point. Although the range of variation was consistent across ecosystems, it had higher absolute amplitudes in grasslands. These differences likely influenced the unique soil conditions and fungal communities observed in forests and grasslands, aligning with findings on the effects of soil‐specific environmental factors on microbial diversity (Hijmans and Elith 2013; De Frenne et al. 2021).

Forest soils, with consistent organic inputs, exhibit tightly coupled properties and stable microclimates, while grasslands show more heterogeneity due to frequent disturbances (De Frenne et al. 2021). This suggests greater resilience in forest soils compared to disturbance‐driven grasslands. In addition, we confirmed that positive correlations between biodiversity and bioclimatic variables such as soil moisture are most pronounced in forests under low LUI, but these relationships weaken or disappear under LUI. For example, Felipe‐Lucia et al. (2020) found that,” at low LUI, strong positive links exist between biodiversity, ecosystem functions, and environmental variables, but these synergies decline as LUI increases, leading to more homogeneous and less integrated ecological networks. This suggests that intensive management disrupts the natural relationships between biodiversity and key environmental factors.

In line with Burst et al. (2020), our results confirm that forests and grasslands host distinct fungal communities, shaped by differences in soil physio‐chemistry and microclimatic conditions. Forests tend to favour fungal specialists due to stable conditions, while grasslands support broader fungal diversity as a result of greater environmental variability and disturbance.

Only a few studies have directly compared soil fungi between these ecosystems, often due to a limited sample size and lacking direct climate measurements (Wang et al. 2023). Using 300 plots with a balanced experimental design across the two ecosystems and various LUI levels, our study provides robust evidence. Similarly, to previous studies, we found higher fungal richness in grasslands compared to forests, underscoring the contrasting ecological dynamics between stable, specialised forest communities and the more diverse, adaptable fungal assemblages in grasslands.

Additionally, fungal β‐diversity patterns reflected differences primarily due to species turnover rather than nestedness. The strong correlation among bioclimatic variables in forests points to unique forest‐specific microhabitats, including the dominance of EMF (Buscot 2015; van der Heijden et al. 2015; Baldrian 2016, 2017; Fernandez and Kennedy 2016). Our results highlight the important role of biotic interactions in shaping fungal communities: EMF dominate in forests, while saprotrophs and AMF prevail in grasslands, reflecting different ecological processes (e.g., decomposition vs. mutualism).

Fungal diversity and composition also differed significantly with LUI, showing ecosystem‐specific trends. In grasslands, we observed increased fungal diversity and evenness with higher LUI—an unexpected pattern that may reflect nutrient‐mediated shifts favouring ruderal taxa under moderate disturbance. As described for fertilised grasslands, where nitrogen enrichment reduces plant diversity but enhances microbial decomposers, increased LUI may similarly promote fungi adapted to resource‐rich environments, albeit at the cost of functional homogenisation (Allison and Martiny 2008; Leff et al. 2015). In forests, however, fungal diversity showed an unimodal response to LUI, with diversity declining at intermediate LUI. This mirrors global observations that moderate disturbance disrupts mycorrhizal networks without facilitating compensatory colonisation (van der Heijden et al. 2015). Forest fungal communities are thus sensitive to fragmentation, beyond which dispersal limitations prevail.

In forests, high LUI was linked to an increase in pathogenic fungi, including *Fusarium*, *Ustilago* and *Alternaria*, which may benefit from altered bioclimatic conditions and diminished EMF presence (Kahl and Bauhus 2014; Mollier et al. 2024). Conversely, in grasslands with high LUI, saprotrophic fungal diversity was reduced, potentially due to soil compaction and lower organic inputs from intensive grazing (Liu et al. 2022). Despite this, overall fungal richness was higher in high LUI grasslands, possibly due to generalist taxa thriving under disturbed conditions (Nacke et al. 2011; Meiser et al. 2014). These patterns indicate that higher LUI levels in grasslands promote biotic homogenisation by favouring generalists over specialists and yielding more uniform communities without necessarily reducing local diversity (Gossner et al. 2016). While this suggests resilience, such homogenisation may limit ecosystem functionality, potentially impairing key services such as decomposition and nutrient cycling (Eisenhauer et al. 2023).

### 4.2 Use of Indicator Species to Predict Ecosystem Resilience to Land Use Changes

Our indicator species analysis identified fungal taxa associated with various LUI levels in each of the ecosystems. In forests, a higher ratio of pathotrophs‐to‐saprotrophs among the indicator species under higher LUI points to a shift toward communities adapted to altered bioclimatic and competitive conditions. In grasslands, high LUI led to a higher saprotroph‐to‐symbiotroph ratio, with bioclimatic stability being a critical factor for these shifts. Indicator species in forests at low LUI were those thriving in stable soil bioclimatic conditions, underscoring their potential as bioindicators of ecosystem health (Goldmann et al. 2015). Our approach thus offers crucial insights into ecosystem resilience (Mollier et al. 2024): the persistent presence of certain fungi under specific conditions allows an assessment of ecosystems differences across land use and bioclimatic gradients. Fungal indicators in forests demonstrate their capacity to maintain functional diversity despite anthropogenic pressures. Conversely, in grasslands, indicator fungi were particularly sensitive to intensified LUI, as shown by shifts in relative abundance linked to soil properties and community changes. The differential sensitivity of these indicator fungi underscores the need to include soil‐specific bioclimatic variables when assessing ecosystem resilience. Changes in their presence may reveal disruptions in key ecosystem functions (e.g., decomposition or nutrient cycling). Consistent with Öpik et al. (2010), our results show that AMF are reliable indicators of stable bioclimatic conditions in grasslands, owing to tight associations with specific host plants. Although symbiont abundances were inferred from ITS sequencing, this approach effectively captured these relevant associations and their ecological significance.

## 5 Conclusion

This study demonstrates that environmental factors, particularly LUI, significantly affect fungal community richness and composition, and highlights the connection between aboveground and belowground ecosystems. While LUI was a primary driver of fungal community differences, general environmental conditions also played a secondary role. Grasslands, given their fungal community structure, appear more sensitive to disturbances than forests, highlighting their vulnerability under rapidly changing climatic and anthropogenic pressures. These results underscore the importance of prioritising targeted management strategies for grasslands to mitigate the impacts of disturbance and promote biodiversity conservation. Future research should further investigate resilience mechanisms in grasslands and forests to refine these strategies and support sustainable ecosystem management.

## Author Contributions

Conceptualization: Rosario Iacono, François Buscot, and Kezia Goldmann. Data curation: Spaska Forteva, Ingo Schöning, Marion Schrumpf, Emily Solly, Stephan Wöllauer, and Kezia Goldmann. Formal analysis: Rosario Iacono. Funding acquisition: François Buscot, Marion Schrumpf, and Kezia Goldmann. Investigation: Ingo Schöning, Marion Schrumpf, and Kezia Goldmann. Methodology: Kezia Goldmann. Project administration: François Buscot and Marion Schrumpf. Supervision: Kezia Goldmann. Visualisation: Rosario Iacono. Writing – original draft: Rosario Iacono and Kezia Goldmann. Writing – review and editing: Rosario Iacono, François Buscot, Spaska Forteva, Ingo Schöning, Emily Solly, Marion Schrumpf, Stephan Wöllauer and Kezia Goldmann.

## Conflicts of Interest

The authors declare no conflicts of interest.

## Supporting information


**Data S1:** emi70170‐sup‐0001‐FigureS1‐S2‐TableS1‐S15.docx.

## Data Availability

This work is based on data collected and elaborated by several projects within the Biodiversity Exploratories programme (DFG Priority Program 1374). All datasets are publicly available in the Biodiversity Exploratories Information System BExIS (http://doi.org/10.17616/R32P9Q): Silvicultural management indices for all the forest plots—BExIS ID 31217 (Schall and Ammer 2024); Soil texture—BExIS ID 14686; pH—data BExIS ID 31074; Total C, total N and total S concentrations—BExIS ID31655; Plant‐available phosphorus—BExIS ID 31340; Climatic data—BExIS ID 24766; Fungal data—BExIS IDs 32062, 32067, 32069. Raw DNA sequences are available on the National Center for Biotechnology Information (NCBI) Sequence Read Archive (SRA) under study accession number PRJNA1213839. The R code used for the data analysis is accessible on GitHub (https://github.com/RosarioIacono/Iacono_et_2025_data_analysis).
