# Supplementary material for: Uncovering the Role of Land Use Intensity in Shaping Forest and Grassland‐Specific Soil Fungal Communities
Source: Environ Microbiol. 2025 Aug 22;27(8):e70170. doi: 10.1111/1462-2920.70170 (PMC12371803; doi:10.1111/1462-2920.70170)
Supplement: Supplementary file 1 — Data S1: emi70170‐sup‐0001‐FigureS1‐S2‐TableS1‐S15.docx. [file EMI-27-e70170-s001.docx]

Uncovering the Role of Land Use Intensity in Shaping Forest and Grassland-Specific Soil Fungal Communities

Rosario Iacono^*^, François Buscot, Spaska Forteva, Ingo Schöning, Marion Schrumpf, Emily Solly, Stephan Wöllauer, and Kezia Goldmann

^*^ Department of Soil Ecology, Helmholtz-Centre for Environmental Research–UFZ, Theodor-Lieser-Straße 4, 06120 Halle (Saale), Germany

E-mail: [rosario.iacono@ufz.de](mailto:rosario.iacono@ufz.de)

# Supplemental Methods

### Details of variable selection for distance based RDA

The method determined the minimum subset of variables necessary to build a model that retains the adjusted R^2^ of the full model. Reducing the number of variables then allowed us to test the significance of the constraints, using an ANOVA permutation test. This test determined which variables were significantly correlated with fungal community differences across ecosystem type and LUI. Analyses were performed on the full dataset as well as separately for forest and grassland. The full db-RDA model included 43 variables and had an adjusted R^2^ of 0.3876. After variable selection, the model was reduced to 26 variables, with an adjusted R^2^ of 0.3872. For the forest and grassland datasets, the models initially also included the same 43 variables, with an adjusted R^2^ of 0.3281 and 0.3325, respectively. After variable selection, the db-RDA for forests had 14 variables (adj. R^2^ = 0.3104), while the grassland model included 13 variables (adj. R^2^ = 0.3271). Although variable reduction resulted in a slight decrease in adjusted R^2^, forward stepwise selection enabled the identification of a minimal, effective variable set. The significance model constraints (environmental factors) was tested using a permutation test for db-RDA.

### Indicator species analysis

In the indicator species method, the INDVAL for each species (i) in each site (j) is defined as the product of two quantities Aij and Bij. Quantity A is the Berger-Parker index (Hill, 1973; Magurran, 1988) and is defined as the mean abundance of the species in the target group divided by the sum of mean abundances across all groups. Quantity B is the Shannon-Weaver exponential index defined as the relative frequency of occurrence of the species inside the target group. INDVAL reaches its maximum when the individuals of a species are observed in all sites of only one group. In the present study, site group was generated matching the ecosystem type (forest or grassland) with three LUI levels (low, medium and high) resulting in six groups.

The hypothesis of a statistically significant association of certain species with certain ecosystem-LUI combinations was tested using the indicator species analysis. Indicator species differ from species association in that they are indicative of particular group of sites.

# Supplemental Figures


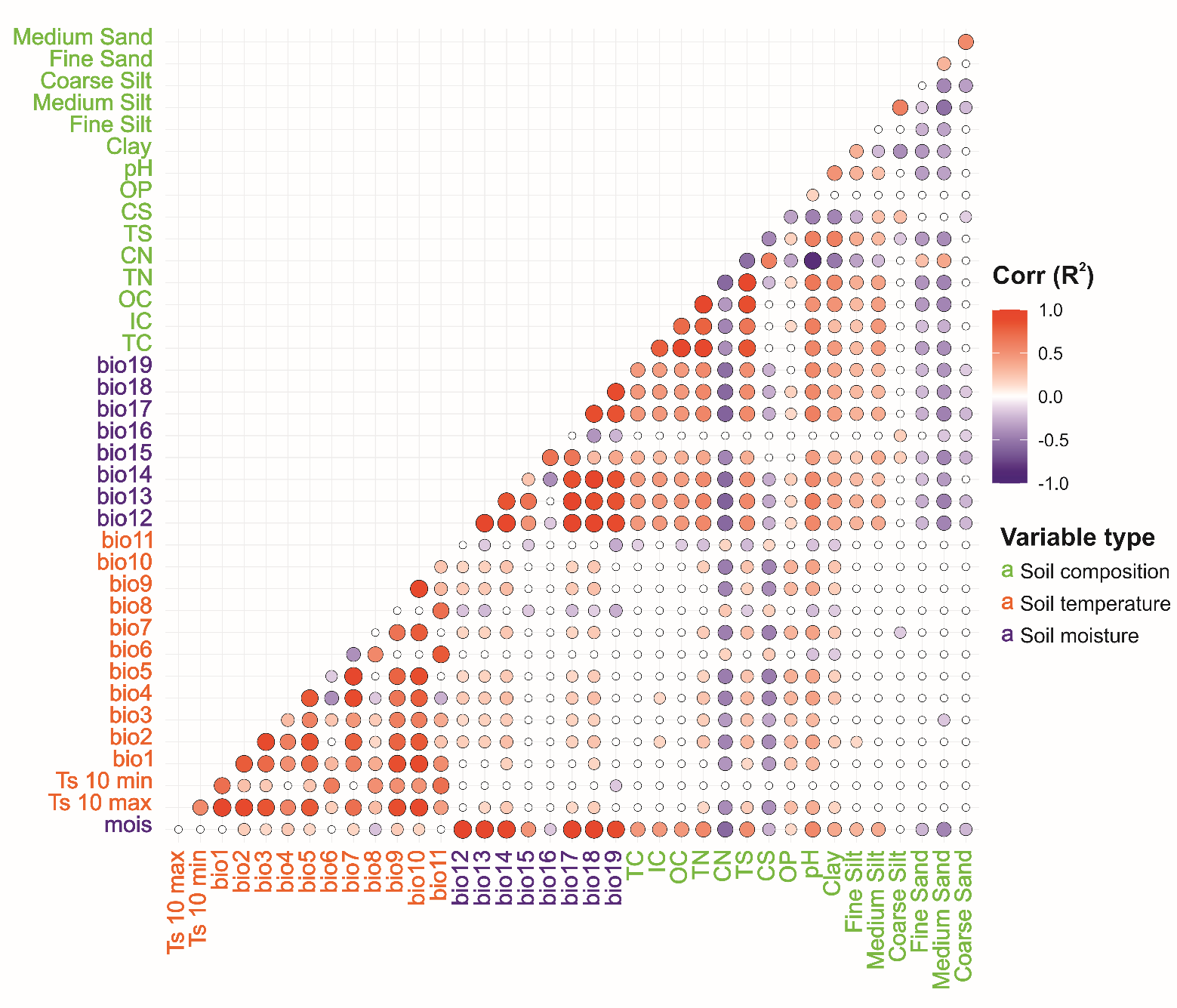


**FIGURE S 1**
Correlation between environmental variables across ecosystems and land use intensity combinations. Correlations were calculated using Pearson correlation method on mixed-effects model residuals, with Bonferroni correction applied for multiple testing. Only significant correlations (adj. p < 0.05) are shown. Color scale shows the correlation coefficient (R2) from 1 (red) to -1 (violet).


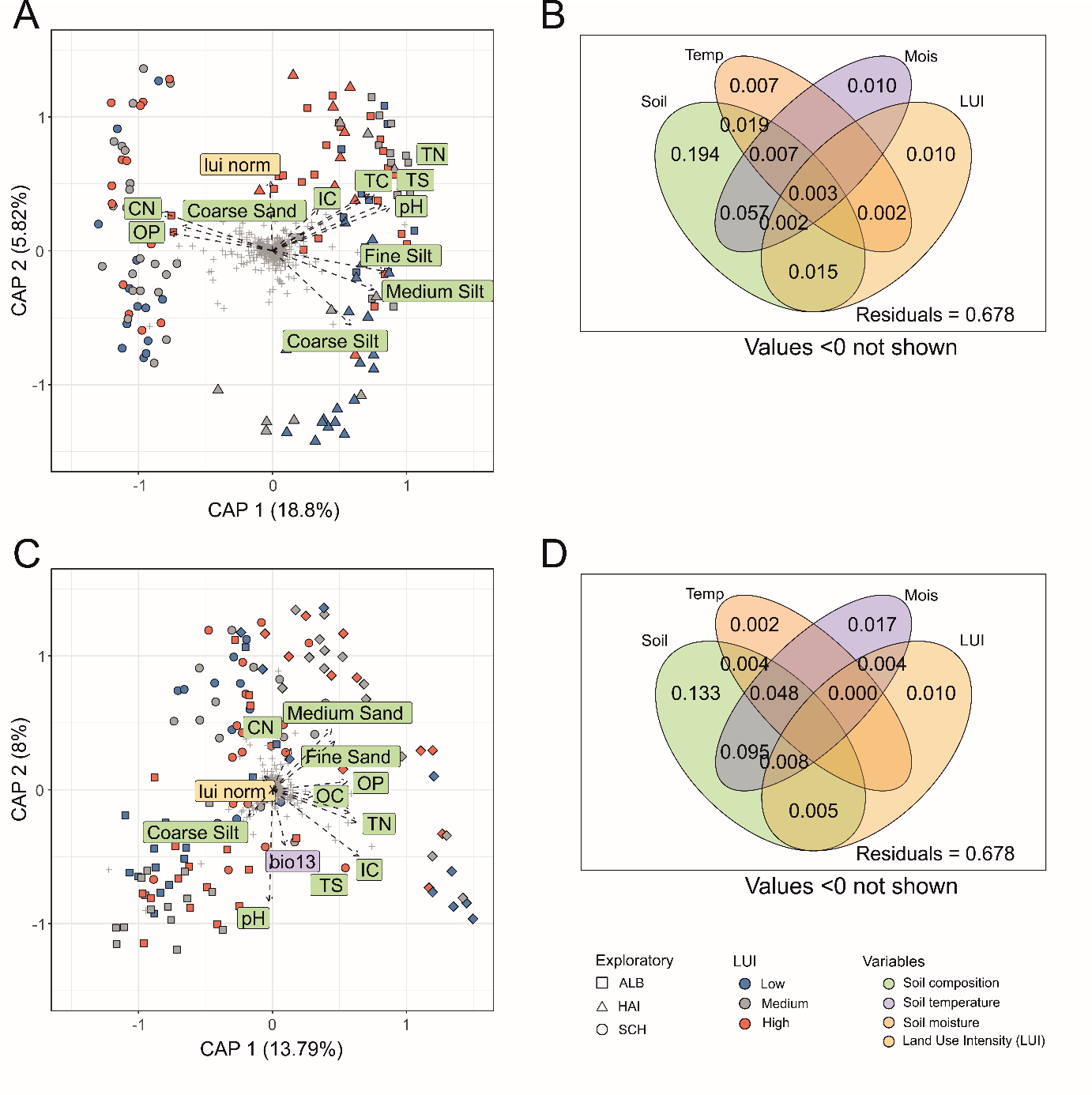


**FIGURE S 2**

Biplots (A and C) and variance partitioning Venn diagrams (B and D) of the distance based redundancy analysis (db-RDA) within forest (A and B) and grassland (C and D).

# Supplemental Tables

***TABLE S 1.*** List of observations from climate stations within the Biodiversity Exploratories used in this work.

| **Name** | **Description** | **Unit** |
| --- | --- | --- |
| SM 10 | Soil moisture at 10 cm below surface | % (percentage of volumetric water content) |
| Ts 10 | Soil temperature at 10 cm below surface | °C (degree Celsius, -40 to 60) |

***TABLE S 2.*** Description of the alpha diversity indices calculated for the fungal communities.

| **α-diversity Index** | **Description** | **Ecological significance** |
| --- | --- | --- |
| N_0_ (q=0) | Species richness | The Number of distinct species in the community (all species weighted equally). |
| N_1_ (q = 1) | Exponential of Shannon entropy | Effective number of common species, weighting species by their frequency. |
| N_2_ (q = 2) | Inverse Simpson diversity | Effective number of dominant species, emphasizing the most abundant species. |
| E_1_ (N_1_/N_0_) | Pielou’s evenness (Shannon-based) | Measures how evenly individuals are distributed among species (0 = uneven, 1 = perfect evenness). |
| E_2_ (N_2_/N_0_) | Simpson evenness | Measures dominance evenness, less sensitive to rare species than E1. |

***TABLE S 3.*** Summary table of the values of environmental variables grouped by ecosystem type (Type) and land use intensity (LUI).

| **Ecosystem** | **Forest** | | | | | | **Grassland** | | | | | |
| --- | --- | --- | --- | --- | --- | --- | --- | --- | --- | --- | --- | --- |
| **LUI** | **Low** | | **Medium** | | **High** | | **Low** | | **Medium** | | **High** | |
| **Variable** | **mean** | **sd** | **mean** | **sd** | **mean** | **sd** | **mean** | **sd** | **mean** | **sd** | **mean** | **sd** |
| mois | 25.71 | 8.69 | 22.46 | 9.58 | 25.90 | 9.88 | 33.58 | 7.16 | 30.88 | 6.79 | 31.77 | 6.50 |
| Ts 10 max | 9.97 | 3.21 | 10.42 | 3.09 | 10.47 | 1.98 | 12.42 | 3.68 | 11.90 | 4.22 | 12.80 | 4.06 |
| Ts 10 min | 6.11 | 1.86 | 6.33 | 1.98 | 6.51 | 1.38 | 6.34 | 2.14 | 6.24 | 2.31 | 6.29 | 1.80 |
| bio1 | 8.04 | 2.42 | 8.38 | 2.42 | 8.49 | 1.63 | 9.38 | 2.77 | 9.07 | 3.22 | 9.55 | 2.82 |
| bio2 | 3.87 | 2.01 | 4.09 | 1.87 | 3.95 | 0.99 | 6.08 | 2.33 | 5.66 | 2.18 | 6.51 | 2.79 |
| bio3 | 23.62 | 6.21 | 24.73 | 7.45 | 24.66 | 5.86 | 28.25 | 7.35 | 27.34 | 6.24 | 30.17 | 6.52 |
| bio4 | 466.14 | 106.73 | 452.83 | 99.89 | 472.86 | 78.38 | 575.17 | 115.91 | 588.68 | 165.39 | 597.24 | 168.86 |
| bio5 | 17.40 | 4.80 | 17.35 | 3.82 | 17.54 | 2.11 | 21.39 | 4.53 | 21.09 | 5.73 | 21.77 | 5.97 |
| bio6 | 1.19 | 1.03 | 1.15 | 1.11 | 1.27 | 1.68 | 0.64 | 1.38 | 0.65 | 0.68 | 0.43 | 1.05 |
| bio7 | 16.22 | 4.73 | 16.19 | 3.83 | 16.27 | 2.81 | 20.75 | 4.97 | 20.44 | 5.58 | 21.34 | 5.97 |
| bio8 | 3.64 | 1.53 | 4.19 | 1.62 | 4.02 | 1.60 | 3.51 | 1.43 | 3.69 | 2.55 | 3.37 | 1.86 |
| bio9 | 11.79 | 4.71 | 12.14 | 5.17 | 13.08 | 3.36 | 15.67 | 5.16 | 14.83 | 6.42 | 16.01 | 4.93 |
| bio10 | 13.74 | 3.17 | 13.84 | 3.41 | 14.33 | 1.89 | 16.72 | 4.24 | 16.66 | 5.16 | 17.31 | 4.59 |
| bio11 | 2.90 | 1.72 | 3.32 | 1.78 | 3.25 | 1.57 | 3.04 | 1.43 | 2.67 | 1.52 | 2.89 | 1.70 |
| bio12 | 25.71 | 8.69 | 22.46 | 9.58 | 25.90 | 9.88 | 33.58 | 7.16 | 30.88 | 6.79 | 31.77 | 6.50 |
| bio13 | 31.66 | 10.19 | 27.14 | 11.82 | 31.04 | 11.43 | 41.00 | 8.38 | 38.94 | 7.93 | 39.22 | 6.86 |
| bio14 | 18.16 | 7.12 | 16.31 | 7.16 | 20.20 | 8.47 | 25.82 | 6.70 | 22.85 | 7.35 | 24.80 | 6.44 |
| bio15 | 13.50 | 5.14 | 10.82 | 6.41 | 10.84 | 4.69 | 15.18 | 5.38 | 16.08 | 6.70 | 14.42 | 2.89 |
| bio16 | 19.70 | 6.45 | 17.02 | 5.50 | 15.91 | 4.88 | 17.15 | 6.04 | 19.75 | 12.04 | 17.29 | 5.16 |
| bio17 | 93.44 | 30.20 | 79.81 | 34.17 | 91.56 | 33.75 | 120.51 | 24.80 | 113.21 | 23.07 | 114.33 | 20.15 |
| bio18 | 58.29 | 21.58 | 52.24 | 22.11 | 63.16 | 25.94 | 81.07 | 20.05 | 72.78 | 21.69 | 77.81 | 19.47 |
| bio19 | 64.41 | 24.69 | 57.29 | 26.23 | 65.74 | 26.82 | 84.77 | 23.63 | 79.99 | 20.22 | 81.95 | 20.56 |
| bio20 | 88.65 | 31.10 | 76.11 | 33.52 | 89.79 | 34.08 | 117.64 | 24.16 | 108.28 | 25.55 | 112.10 | 22.01 |
| TC | 39.55 | 19.09 | 40.31 | 20.10 | 52.18 | 21.89 | 105.04 | 85.68 | 77.19 | 71.22 | 75.82 | 63.16 |
| IC | 0.27 | 0.43 | 0.43 | 1.22 | 0.53 | 0.97 | 19.18 | 25.09 | 6.75 | 15.20 | 4.04 | 11.65 |
| OC | 39.28 | 18.79 | 39.88 | 19.63 | 51.65 | 21.34 | 85.86 | 72.62 | 70.44 | 66.94 | 71.78 | 60.67 |
| TN | 2.86 | 1.56 | 2.77 | 1.75 | 3.63 | 1.71 | 8.14 | 6.20 | 6.61 | 5.37 | 6.87 | 4.88 |
| CN | 14.58 | 2.53 | 15.96 | 3.44 | 15.04 | 2.69 | 10.44 | 0.82 | 10.37 | 1.02 | 10.08 | 0.70 |
| TS | 0.31 | 0.18 | 0.32 | 0.19 | 0.40 | 0.18 | 0.75 | 0.47 | 0.85 | 0.93 | 1.01 | 0.94 |
| CS | 133.44 | 16.14 | 133.85 | 24.49 | 133.00 | 16.90 | 112.62 | 55.49 | 92.03 | 39.47 | 74.57 | 15.34 |
| OP | 10.63 | 11.31 | 13.06 | 11.70 | 9.60 | 9.46 | 16.42 | 14.98 | 17.31 | 10.50 | 19.63 | 10.29 |
| pH | 4.50 | 0.83 | 4.50 | 1.17 | 4.83 | 1.06 | 6.81 | 0.59 | 6.65 | 0.58 | 6.56 | 0.62 |
| Clay | 27.23 | 18.48 | 23.24 | 20.71 | 32.91 | 21.24 | 33.65 | 17.32 | 36.91 | 21.52 | 42.69 | 17.87 |
| Fine Silt | 8.42 | 4.35 | 5.96 | 4.42 | 8.55 | 4.81 | 8.99 | 5.79 | 9.50 | 5.84 | 10.08 | 4.99 |
| Medium Silt | 15.97 | 9.56 | 12.09 | 12.00 | 14.23 | 8.91 | 23.01 | 14.89 | 18.02 | 16.27 | 15.46 | 9.50 |
| Coarse Silt | 20.32 | 12.31 | 15.16 | 12.77 | 15.36 | 9.71 | 15.85 | 6.54 | 14.03 | 7.34 | 14.94 | 8.18 |
| Fine Sand | 11.61 | 12.05 | 15.50 | 15.51 | 8.76 | 10.52 | 9.01 | 7.61 | 9.02 | 9.00 | 5.61 | 5.50 |
| Medium Sand | 13.96 | 22.09 | 22.61 | 24.62 | 13.79 | 21.74 | 7.67 | 10.88 | 9.59 | 14.01 | 8.22 | 14.60 |
| Coarse Sand | 2.57 | 4.49 | 5.51 | 8.01 | 4.44 | 8.58 | 1.82 | 2.26 | 2.96 | 4.44 | 3.01 | 5.28 |

***TABLE S 4.*** Result of the ANOVA test of the mixed linear model fit for each variable with Exploratory as a random effect. Ecosystem type (Type) and Land use intensity (LUI) are considered fixed effects. Values with p<0.05 are considered statistically significant (bold).

| **Variable** | **Factor** | **Sum Sq** | **Mean Sq** | **NumDF** | **DenDF** | **F value** | **Pr(>F)** |
| --- | --- | --- | --- | --- | --- | --- | --- |
| mois | Type | 4098.198 | 4098.198 | 1 | 292 | 104.377 | **0.000** |
|  | LUI | 426.569 | 213.285 | 2 | 292.148 | 5.432 | **0.005** |
|  | Type:LUI | 118.601 | 59.301 | 2 | 292.102 | 1.51 | 0.223 |
| Ts 10 max | Type | 326.575 | 326.575 | 1 | 294 | 27.377 | 0.000 |
|  | LUI | 13.819 | 6.91 | 2 | 294 | 0.579 | 0.561 |
|  | Type:LUI | 13.893 | 6.946 | 2 | 294 | 0.582 | 0.559 |
| Ts 10 min | Type | 0.052 | 0.052 | 1 | 292.007 | 0.014 | 0.906 |
|  | LUI | 4.105 | 2.053 | 2 | 293.977 | 0.561 | 0.571 |
|  | Type:LUI | 2.387 | 1.193 | 2 | 293.225 | 0.326 | 0.722 |
|  | LUI | 664.957 | 332.479 | 2 | 294 | 2.048 | 0.131 |
|  | Type:LUI | 664.957 | 332.479 | 2 | 294 | 2.048 | 0.131 |
| bio1 | Type | 79.599 | 79.599 | 1 | 292.014 | 11.888 | **0.001** |
|  | LUI | 8.144 | 4.072 | 2 | 290.838 | 0.608 | 0.545 |
|  | Type:LUI | 4.691 | 2.346 | 2 | 290.455 | 0.35 | 0.705 |
| bio2 | Type | 334.826 | 334.826 | 1 | 294 | 75.726 | **0.000** |
|  | LUI | 6.777 | 3.389 | 2 | 294 | 0.766 | 0.466 |
|  | Type:LUI | 12.641 | 6.32 | 2 | 294 | 1.429 | 0.241 |
| bio3 | Type | 1315.186 | 1315.186 | 1 | 284.01 | 30.247 | **0.000** |
|  | LUI | 85.94 | 42.97 | 2 | 285.546 | 0.988 | 0.374 |
|  | Type:LUI | 116.003 | 58.001 | 2 | 284.745 | 1.334 | 0.265 |
| bio4 | Type | 1136309.502 | 1136310 | 1 | 291.992 | 71.847 | **0.000** |
|  | LUI | 27476.791 | 13738.4 | 2 | 293.993 | 0.869 | 0.421 |
|  | Type:LUI | 6862.273 | 3431.137 | 2 | 293.199 | 0.217 | 0.805 |
| bio5 | Type | 1192.189 | 1192.189 | 1 | 291.987 | 54.946 | **0.000** |
|  | LUI | 14.378 | 7.189 | 2 | 291.675 | 0.331 | 0.718 |
|  | Type:LUI | 2.182 | 1.091 | 2 | 291.127 | 0.05 | 0.951 |
| bio6 | Type | 29.878 | 29.878 | 1 | 291.993 | 21.145 | **0.000** |
|  | LUI | 0.416 | 0.208 | 2 | 293.94 | 0.147 | 0.863 |
|  | Type:LUI | 1.268 | 0.634 | 2 | 293.114 | 0.449 | 0.639 |
| bio7 | Type | 1599.541 | 1599.541 | 1 | 291.986 | 71.241 | **0.000** |
|  | LUI | 20.199 | 10.1 | 2 | 293.58 | 0.45 | 0.638 |
|  | Type:LUI | 6.254 | 3.127 | 2 | 292.75 | 0.139 | 0.870 |
| bio8 | Type | 13.913 | 13.913 | 1 | 292.009 | 4.385 | **0.037** |
|  | LUI | 5.386 | 2.693 | 2 | 293.931 | 0.849 | 0.429 |
|  | Type:LUI | 2.031 | 1.016 | 2 | 293.217 | 0.32 | 0.726 |
| bio9 | Type | 751.491 | 751.491 | 1 | 294 | 29.601 | **0.000** |
|  | LUI | 61.457 | 30.729 | 2 | 294 | 1.21 | 0.300 |
|  | Type:LUI | 19.785 | 9.892 | 2 | 294 | 0.39 | 0.678 |
| bio10 | Type | 641.338 | 641.338 | 1 | 292.005 | 42.676 | **0.000** |
|  | LUI | 30.696 | 15.348 | 2 | 292.482 | 1.021 | 0.361 |
|  | Type:LUI | 0.271 | 0.135 | 2 | 291.806 | 0.009 | 0.991 |
| bio11 | Type | 6.229 | 6.229 | 1 | 292.025 | 2.369 | 0.125 |
|  | LUI | 0.794 | 0.397 | 2 | 290.826 | 0.151 | 0.860 |
|  | Type:LUI | 7.014 | 3.507 | 2 | 290.45 | 1.334 | 0.265 |
| bio12 | Type | 4098.169 | 4098.169 | 1 | 292 | 104.377 | **0.000** |
|  | LUI | 426.569 | 213.285 | 2 | 292.148 | 5.432 | **0.005** |
|  | Type:LUI | 118.599 | 59.3 | 2 | 292.102 | 1.51 | 0.223 |
| bio13 | Type | 7163.678 | 7163.678 | 1 | 292 | 129.658 | **0.000** |
|  | LUI | 494.21 | 247.105 | 2 | 292.158 | 4.472 | **0.012** |
|  | Type:LUI | 137.575 | 68.788 | 2 | 292.108 | 1.245 | 0.289 |
| bio14 | Type | 2945.829 | 2945.829 | 1 | 292 | 94.961 | **0.000** |
|  | LUI | 259.406 | 129.703 | 2 | 292.154 | 4.181 | **0.016** |
|  | Type:LUI | 155.879 | 77.939 | 2 | 292.106 | 2.512 | 0.083 |
| bio15 | Type | 921.899 | 921.899 | 1 | 292.001 | 37.562 | **0.000** |
|  | LUI | 143.741 | 71.871 | 2 | 292.579 | 2.928 | **0.055** |
|  | Type:LUI | 66.386 | 33.193 | 2 | 292.393 | 1.352 | 0.260 |
| bio16 | Type | 19.869 | 19.869 | 1 | 291.997 | 0.422 | 0.516 |
|  | LUI | 87.688 | 43.844 | 2 | 293.095 | 0.932 | 0.395 |
|  | Type:LUI | 274.072 | 137.036 | 2 | 292.731 | 2.912 | 0.056 |
| bio17 | Type | 57747.613 | 57747.61 | 1 | 292 | 120.277 | **0.000** |
|  | LUI | 4841.806 | 2420.903 | 2 | 292.162 | 5.042 | **0.007** |
|  | Type:LUI | 1385.387 | 692.694 | 2 | 292.111 | 1.443 | 0.238 |
| bio18 | Type | 28009.096 | 28009.1 | 1 | 292 | 96.728 | **0.000** |
|  | LUI | 2383.939 | 1191.97 | 2 | 292.161 | 4.116 | **0.017** |
|  | Type:LUI | 1075.837 | 537.918 | 2 | 292.11 | 1.858 | 0.158 |
| bio19 | Type | 29274.738 | 29274.74 | 1 | 292 | 83.479 | **0.000** |
|  | LUI | 2289.776 | 1144.888 | 2 | 292.171 | 3.265 | **0.040** |
|  | Type:LUI | 360.315 | 180.158 | 2 | 292.117 | 0.514 | 0.599 |
| bio20 | Type | 58067.244 | 58067.24 | 1 | 292 | 116.74 | 0.000 |
|  | LUI | 4566.704 | 2283.352 | 2 | 292.159 | 4.591 | 0.011 |
|  | Type:LUI | 1809.791 | 904.895 | 2 | 292.109 | 1.819 | 0.164 |
| bio21 | Type | 0.436 | 0.436 | 1 | 294 | 21.343 | **0.000** |
|  | LUI | 0.026 | 0.013 | 2 | 294 | 0.634 | 0.531 |
|  | Type:LUI | 0.026 | 0.013 | 2 | 294 | 0.634 | 0.531 |
| bio22 | Type | 1.929 | 1.929 | 1 | 294 | 21.743 | **0.000** |
|  | LUI | 0.081 | 0.04 | 2 | 294 | 0.456 | 0.634 |
|  | Type:LUI | 0.081 | 0.04 | 2 | 294 | 0.456 | 0.634 |
| bio23 | Type | 0.004 | 0.004 | 1 | 291.997 | 15.446 | **0.000** |
|  | LUI | 0.001 | 0 | 2 | 290.494 | 1.543 | 0.215 |
|  | Type:LUI | 0.001 | 0.001 | 2 | 290.167 | 1.932 | 0.147 |
| bio24 | Type | 1.75 | 1.75 | 1 | 294 | 21.767 | **0.000** |
|  | LUI | 0.066 | 0.033 | 2 | 294 | 0.409 | 0.665 |
|  | Type:LUI | 0.066 | 0.033 | 2 | 294 | 0.409 | 0.665 |
| TC | Type | 132325.632 | 132325.6 | 1 | 291.999 | 47.459 | 0.000 |
|  | LUI | 14285.594 | 7142.797 | 2 | 293.368 | 2.562 | 0.079 |
|  | Type:LUI | 12818.228 | 6409.114 | 2 | 292.905 | 2.299 | 0.102 |
| IC | Type | 6879.974 | 6879.974 | 1 | 292.004 | 44.279 | **0.000** |
|  | LUI | 2918.323 | 1459.162 | 2 | 293.156 | 9.391 | **0.000** |
|  | Type:LUI | 2608.61 | 1304.305 | 2 | 292.772 | 8.394 | **0.000** |
| OC | Type | 78861.658 | 78861.66 | 1 | 291.997 | 33.755 | **0.000** |
|  | LUI | 5409.493 | 2704.746 | 2 | 293.569 | 1.158 | 0.316 |
|  | Type:LUI | 4099.801 | 2049.9 | 2 | 293.026 | 0.877 | 0.417 |
| TN | Type | 1274.028 | 1274.028 | 1 | 291.996 | 80.434 | **0.000** |
|  | LUI | 54.417 | 27.208 | 2 | 293.51 | 1.718 | 0.181 |
|  | Type:LUI | 22.849 | 11.425 | 2 | 292.99 | 0.721 | 0.487 |
| CN | Type | 1799.721 | 1799.721 | 1 | 292.001 | 579.711 | **0.000** |
|  | LUI | 14.626 | 7.313 | 2 | 292.216 | 2.356 | 0.097 |
|  | Type:LUI | 9.537 | 4.769 | 2 | 292.148 | 1.536 | 0.217 |
| TS | Type | 20.782 | 20.782 | 1 | 291.986 | 60.647 | **0.000** |
|  | LUI | 1.234 | 0.617 | 2 | 293.835 | 1.8 | 0.167 |
|  | Type:LUI | 0.556 | 0.278 | 2 | 292.995 | 0.812 | 0.445 |
| CS | Type | 122151.55 | 122151.6 | 1 | 292.001 | 154.756 | **0.000** |
|  | LUI | 13742.915 | 6871.457 | 2 | 292.375 | 8.706 | **0.000** |
|  | Type:LUI | 12109.767 | 6054.883 | 2 | 292.256 | 7.671 | **0.001** |
| OP | Type | 3359.584 | 3359.584 | 1 | 292 | 43.576 | **0.000** |
|  | LUI | 341.715 | 170.857 | 2 | 292.148 | 2.216 | 0.111 |
|  | Type:LUI | 725.725 | 362.862 | 2 | 292.101 | 4.707 | **0.010** |
| pH | Type | 319.218 | 319.218 | 1 | 292.001 | 579.073 | **0.000** |
|  | LUI | 0.02 | 0.01 | 2 | 292.361 | 0.018 | 0.982 |
|  | Type:LUI | 7.354 | 3.677 | 2 | 292.246 | 6.67 | **0.001** |
| Clay | Type | 7434.145 | 7434.145 | 1 | 292 | 65.552 | **0.000** |
|  | LUI | 80.462 | 40.231 | 2 | 292.045 | 0.355 | 0.702 |
|  | Type:LUI | 104.711 | 52.355 | 2 | 292.031 | 0.462 | 0.631 |
| Fine Silt | Type | 265.644 | 265.644 | 1 | 292 | 23.053 | **0.000** |
|  | LUI | 9.022 | 4.511 | 2 | 292.088 | 0.391 | 0.676 |
|  | Type:LUI | 32.073 | 16.037 | 2 | 292.06 | 1.392 | 0.250 |
| Medium Silt | Type | 1681.28 | 1681.28 | 1 | 292.002 | 12.825 | **0.000** |
|  | LUI | 1287.157 | 643.579 | 2 | 292.705 | 4.909 | **0.008** |
|  | Type:LUI | 835.959 | 417.979 | 2 | 292.477 | 3.188 | **0.043** |
| Coarse Silt | Type | 302.405 | 302.405 | 1 | 292 | 5.951 | **0.015** |
|  | LUI | 280.372 | 140.186 | 2 | 292.121 | 2.759 | 0.065 |
|  | Type:LUI | 27.302 | 13.651 | 2 | 292.082 | 0.269 | 0.765 |
| Fine Sand | Type | 1246.441 | 1246.441 | 1 | 292 | 27.454 | **0.000** |
|  | LUI | 212.399 | 106.2 | 2 | 292.075 | 2.339 | 0.098 |
|  | Type:LUI | 82.378 | 41.189 | 2 | 292.051 | 0.907 | 0.405 |
| Medium Sand | Type | 5160.113 | 5160.113 | 1 | 292 | 58.993 | **0.000** |
|  | LUI | 784.746 | 392.373 | 2 | 292.036 | 4.486 | **0.012** |
|  | Type:LUI | 703.295 | 351.647 | 2 | 292.025 | 4.02 | **0.019** |
| Coarse Sand | Type | 186.914 | 186.914 | 1 | 292 | 10.432 | **0.001** |
|  | LUI | 321.644 | 160.822 | 2 | 292.112 | 8.976 | **0.000** |
|  | Type:LUI | 7.874 | 3.937 | 2 | 292.077 | 0.22 | 0.803 |

**TABLE S 5.** Estimated marginal means (emm) for mixed linear models. Significant differences were corrected using multivariate t distribution and are reported in a compact letter display (cld). Different letters represent significantly different groups with a p<0.01. Degrees of freedom (df) were calculated using Satterthwaite method.

| **Variable** | **Forest** | | | | | | | | | **Grassland** | | | | | | | | |
| --- | --- | --- | --- | --- | --- | --- | --- | --- | --- | --- | --- | --- | --- | --- | --- | --- | --- | --- |
|  | **Low** | | | **Medium** | | | **High** | | | **Low** | | | **Medium** | | | **High** | | |
|  | **emm** | **SE** | **cld** | **emm** | **SE** | **cld** | **emm** | **SE** | **cld** | **emm** | **SE** | **Cld** | **emm** | **SE** | **cld** | **emm** | **SE** | **cld** |
| **mois** | 25.618 | 3.887 | c | 23.772 | 3.884 | c | 24.675 | 3.886 | c | 34.567 | 3.883 | a | 31.178 | 3.883 | ab | 30.496 | 3.884 | b |
| **Ts 10 max** | 9.974 | 0.488 | c | 10.423 | 0.488 | c | 10.468 | 0.488 | bc | 12.420 | 0.488 | ab | 11.905 | 0.488 | abc | 12.801 | 0.488 | a |
| **Ts 10 min** | 6.092 | 0.345 | a | 6.272 | 0.343 | a | 6.589 | 0.344 | a | 6.294 | 0.343 | a | 6.230 | 0.342 | a | 6.350 | 0.343 | a |
| **bio1** | 8.026 | 0.393 | b | 8.351 | 0.392 | ab | 8.532 | 0.393 | ab | 9.358 | 0.391 | ab | 9.068 | 0.391 | ab | 9.574 | 0.391 | a |
| **bio2** | 3.865 | 0.297 | b | 4.094 | 0.297 | b | 3.954 | 0.297 | b | 6.079 | 0.297 | a | 5.661 | 0.297 | a | 6.512 | 0.297 | a |
| **bio3** | 23.756 | 1.087 | c | 24.831 | 1.082 | bc | 24.444 | 1.077 | c | 28.339 | 1.071 | ab | 27.384 | 1.096 | abc | 30.046 | 1.098 | a |
| **bio4** | 460.479 | 22.134 | b | 452.215 | 22.011 | b | 479.135 | 22.089 | b | 573.677 | 21.971 | a | 588.331 | 21.941 | a | 599.086 | 21.993 | a |
| **bio5** | 17.313 | 0.717 | b | 17.331 | 0.714 | b | 17.647 | 0.716 | b | 21.360 | 0.713 | a | 21.085 | 0.712 | a | 21.807 | 0.713 | a |
| **bio6** | 1.245 | 0.203 | a | 1.126 | 0.202 | ab | 1.243 | 0.203 | a | 0.628 | 0.202 | ab | 0.647 | 0.201 | ab | 0.445 | 0.202 | b |
| **bio7** | 16.046 | 0.774 | b | 16.197 | 0.770 | b | 16.435 | 0.773 | b | 20.722 | 0.769 | a | 20.436 | 0.768 | a | 21.374 | 0.770 | a |
| **bio8** | 3.678 | 0.329 | a | 4.121 | 0.328 | a | 4.057 | 0.329 | a | 3.458 | 0.327 | a | 3.676 | 0.327 | a | 3.429 | 0.327 | a |
| **bio9** | 11.786 | 0.713 | d | 12.140 | 0.713 | cd | 13.081 | 0.713 | bcd | 15.666 | 0.713 | ab | 14.832 | 0.713 | abc | 16.006 | 0.713 | a |
| **bio10** | 13.689 | 0.607 | c | 13.803 | 0.604 | c | 14.421 | 0.605 | bc | 16.680 | 0.603 | a | 16.648 | 0.602 | ab | 17.358 | 0.603 | a |
| **bio11** | 2.911 | 0.246 | a | 3.298 | 0.245 | a | 3.263 | 0.246 | a | 3.028 | 0.245 | a | 2.670 | 0.245 | a | 2.909 | 0.245 | a |
| **bio12** | 25.618 | 3.887 | c | 23.772 | 3.884 | c | 24.675 | 3.886 | c | 34.567 | 3.883 | a | 31.178 | 3.883 | ab | 30.496 | 3.884 | b |
| **bio13** | 31.211 | 4.472 | c | 28.733 | 4.469 | c | 29.894 | 4.471 | c | 42.133 | 4.468 | a | 39.279 | 4.468 | ab | 37.745 | 4.469 | b |
| **bio14** | 18.390 | 3.387 | c | 17.358 | 3.385 | c | 18.926 | 3.387 | c | 26.657 | 3.385 | a | 23.099 | 3.384 | b | 23.718 | 3.385 | ab |
| **bio15** | 12.842 | 1.611 | bcd | 11.359 | 1.607 | cd | 10.963 | 1.609 | d | 15.467 | 1.606 | ab | 16.177 | 1.605 | a | 14.038 | 1.607 | abc |
| **bio16** | 18.995 | 1.676 | a | 17.003 | 1.670 | a | 16.642 | 1.673 | a | 17.001 | 1.668 | a | 19.714 | 1.666 | a | 17.470 | 1.669 | a |
| **bio17** | 92.009 | 13.031 | c | 84.479 | 13.023 | c | 88.313 | 13.028 | c | 123.800 | 13.020 | a | 114.215 | 13.018 | ab | 110.031 | 13.021 | b |
| **bio18** | 59.053 | 10.139 | c | 55.332 | 10.132 | c | 59.303 | 10.137 | c | 83.564 | 10.130 | a | 73.510 | 10.129 | b | 74.589 | 10.132 | ab |
| **bio19** | 65.183 | 10.849 | b | 60.608 | 10.842 | b | 61.650 | 10.846 | b | 87.439 | 10.839 | a | 80.766 | 10.837 | a | 78.506 | 10.840 | a |
| **bio20** | 87.441 | 13.360 | c | 80.841 | 13.351 | c | 86.264 | 13.357 | c | 121.024 | 13.348 | a | 109.307 | 13.346 | ab | 107.690 | 13.350 | b |
| **TC** | 44.314 | 11.686 | c | 37.516 | 11.637 | c | 50.213 | 11.668 | bc | 103.782 | 11.621 | a | 76.738 | 11.609 | ab | 77.534 | 11.630 | ab |
| **IC** | 0.741 | 2.976 | b | -0.445 | 2.965 | b | 0.936 | 2.972 | b | 18.596 | 2.961 | a | 6.568 | 2.959 | b | 4.801 | 2.963 | b |
| **OC** | 43.464 | 10.039 | cd | 37.993 | 9.993 | d | 49.353 | 10.022 | bcd | 85.191 | 9.977 | a | 70.173 | 9.966 | abc | 72.725 | 9.986 | ab |
| **TN** | 3.226 | 0.842 | b | 2.630 | 0.838 | b | 3.407 | 0.840 | b | 8.098 | 0.837 | a | 6.595 | 0.836 | a | 6.934 | 0.837 | a |
| **CN** | 14.667 | 0.911 | a | 15.636 | 0.911 | a | 15.275 | 0.911 | a | 10.211 | 0.910 | b | 10.298 | 0.910 | b | 10.374 | 0.911 | b |
| **TS** | 0.335 | 0.098 | b | 0.312 | 0.097 | b | 0.382 | 0.098 | b | 0.751 | 0.097 | a | 0.847 | 0.097 | a | 1.010 | 0.097 | a |
| **CS** | 135.447 | 11.173 | a | 129.888 | 11.157 | a | 134.959 | 11.167 | a | 109.967 | 11.152 | b | 91.212 | 11.148 | c | 78.044 | 11.155 | c |
| **OP** | 11.769 | 5.448 | c | 11.013 | 5.445 | c | 10.505 | 5.447 | c | 15.072 | 5.444 | bc | 16.893 | 5.443 | ab | 21.400 | 5.445 | a |
| **pH** | 4.423 | 0.301 | b | 4.614 | 0.300 | b | 4.793 | 0.300 | b | 6.877 | 0.300 | a | 6.674 | 0.300 | a | 6.468 | 0.300 | a |
| **Clay** | 28.350 | 11.835 | b | 26.869 | 11.833 | b | 28.160 | 11.835 | b | 36.612 | 11.832 | a | 37.773 | 11.832 | a | 38.863 | 11.833 | a |
| **Fine Silt** | 7.870 | 2.723 | ab | 6.993 | 2.723 | b | 8.061 | 2.723 | ab | 9.678 | 2.722 | a | 9.711 | 2.722 | a | 9.181 | 2.722 | a |
| **Medium Silt** | 14.959 | 3.407 | b | 13.236 | 3.398 | b | 14.091 | 3.404 | b | 23.697 | 3.395 | a | 18.245 | 3.393 | ab | 14.548 | 3.397 | b |
| **Coarse Silt** | 17.858 | 4.888 | A | 16.873 | 4.886 | a | 16.107 | 4.888 | a | 16.706 | 4.885 | a | 14.322 | 4.885 | a | 13.786 | 4.886 | a |
| **Fine Sand** | 12.370 | 5.846 | ab | 13.343 | 5.844 | a | 10.153 | 5.845 | abc | 7.509 | 5.843 | c | 8.556 | 5.843 | bc | 7.570 | 5.843 | c |
| **Medium Sand** | 16.005 | 11.600 | ab | 18.207 | 11.598 | a | 16.152 | 11.599 | ab | 4.694 | 11.598 | d | 8.668 | 11.597 | cd | 12.118 | 11.598 | bc |
| **Coarse Sand** | 2.970 | 3.012 | ab | 4.407 | 3.011 | a | 5.146 | 3.012 | a | 1.054 | 3.010 | b | 2.721 | 3.010 | ab | 4.013 | 3.011 | a |

**TABLE S 6.** Summary table of the fungal diversity indices in the two ecosystems (Forest or Grassland) and under the land use intensity levels (Low, Medium, High).

| Ecosystem | **Forest** | | | | | | **Grassland** | | | | | |
| --- | --- | --- | --- | --- | --- | --- | --- | --- | --- | --- | --- | --- |
| LUI | Low | | Medium | | High | | Low | | Medium | | High | |
| Stat | mean | sd | mean | sd | mean | sd | mean | sd | mean | sd | mean | sd |
| n spec | 316.84 | 56.75 | 301.88 | 69.6 | 326.54 | 67.69 | 340.9 | 43.5 | 353 | 51.93 | 355.64 | 45.5 |
| N1 | 88.62 | 27.21 | 81.61 | 29.52 | 89.71 | 24.2 | 86.85 | 19.56 | 89.89 | 22.77 | 93.42 | 23.89 |
| N2 | 42.63 | 15.66 | 38.38 | 17.11 | 42.62 | 13.65 | 36.3 | 11.43 | 39.43 | 14.35 | 41.13 | 15.54 |
| E1 | 0.28 | 0.05 | 0.26 | 0.04 | 0.27 | 0.04 | 0.25 | 0.04 | 0.25 | 0.05 | 0.26 | 0.05 |
| E2 | 0.13 | 0.04 | 0.12 | 0.04 | 0.13 | 0.03 | 0.11 | 0.03 | 0.11 | 0.04 | 0.11 | 0.04 |

**TABLE S 7.** Result of the ANOVA test of the mixed linear model for the effect of ecosystem type (Type) and Land Use Intensity (LUI) on the fungal diversity indices calculated.

| **Variable** | **Factor** | **Sum Sq** | **Mean Sq** | **NumDF** | **DenDF** | **F value** | **Pr(>F)** |
| --- | --- | --- | --- | --- | --- | --- | --- |
| N_0_ (q = 0) | Type | 90619.314 | 90619.314 | 1 | 292.001 | 30.01 | 0 |
|  | LUI | 3727.347 | 1863.673 | 2 | 293.221 | 0.617 | 0.54 |
|  | Type:LUI | 8790.659 | 4395.33 | 2 | 292.813 | 1.456 | 0.235 |
| N_1_ (q = 1) | Type | 868.596 | 868.596 | 1 | 292.007 | 1.467 | 0.227 |
|  | LUI | 897.773 | 448.887 | 2 | 293.813 | 0.758 | 0.469 |
|  | Type:LUI | 877.92 | 438.96 | 2 | 293.165 | 0.741 | 0.477 |
| N_2_ (q = 2) | Type | 382.415 | 382.415 | 1 | 292.015 | 1.785 | 0.183 |
|  | LUI | 339.779 | 169.889 | 2 | 293.721 | 0.793 | 0.453 |
|  | Type:LUI | 568.075 | 284.038 | 2 | 292.896 | 1.326 | 0.267 |
| E_1_ (N_1_/N_0_) | Type | 0.018 | 0.018 | 1 | 291.998 | 8.584 | 0.004 |
|  | LUI | 0.003 | 0.001 | 2 | 293.794 | 0.624 | 0.536 |
|  | Type:LUI | 0.001 | 0 | 2 | 292.959 | 0.119 | 0.888 |
| E_2_ (N_2_/N_0_) | Type | 0.027 | 0.027 | 1 | 291.976 | 21.059 | 0 |
|  | LUI | 0.001 | 0.001 | 2 | 289.174 | 0.459 | 0.633 |
|  | Type:LUI | 0.002 | 0.001 | 2 | 289.093 | 0.763 | 0.467 |

**Table S 8.** Estimated marginal means (emm) and posthoc test results of the mixed linear model for the effect of ecosystem type and land use intensity on fungal diversity indices. Column “cld” contains the compact letter display presentation of statistical differences.

|  |  | **Land Use Intensity** | | | | | | | | | | | |
| --- | --- | --- | --- | --- | --- | --- | --- | --- | --- | --- | --- | --- | --- |
|  | **var** | **Low** | | | | **Medium** | | | | **High** | | | |
|  |  | **emm** | **SE** | **df** | **cld** | **emm** | **SE** | **df** | **cld** | **emm** | **SE** | **df** | **cld** |
| **Forest** | N_0_ | 319.451 | 12.796 | 4.263 | cd | 304.059 | 12.746 | 4.208 | d | 321.75 | 12.778 | 4.243 | bcd |
|  | N_1_ | 88.54 | 4.695 | 6.757 | a | 82.611 | 4.671 | 6.674 | a | 88.798 | 4.686 | 6.726 | a |
|  | N_2_ | 42.5 | 2.419 | 13.225 | a | 38.75 | 2.405 | 13.324 | a | 42.38 | 2.414 | 13.261 | a |
|  | E_1_ | 0.273 | 0.008 | 12.541 | a | 0.266 | 0.008 | 12.605 | a | 0.274 | 0.008 | 12.564 | a |
|  | E_2_ | 0.132 | 0.005 | 25.828 | a | 0.125 | 0.005 | 27.885 | abc | 0.131 | 0.005 | 26.558 | ab |
| **Grassland** | N_0_ | 343.035 | 12.73 | 4.19 | abc | 353.592 | 12.719 | 4.177 | a | 352.913 | 12.739 | 4.2 | ab |
|  | N_1_ | 87.593 | 4.663 | 6.646 | a | 90.114 | 4.657 | 6.626 | a | 92.45 | 4.667 | 6.662 | a |
|  | N_2_ | 36.556 | 2.401 | 13.354 | a | 39.507 | 2.398 | 13.376 | a | 40.794 | 2.403 | 13.338 | a |
|  | E_1_ | 0.254 | 0.008 | 12.624 | a | 0.253 | 0.008 | 12.638 | a | 0.259 | 0.008 | 12.614 | a |
|  | E_2_ | 0.106 | 0.005 | 28.612 | c | 0.111 | 0.005 | 29.156 | bc | 0.114 | 0.005 | 28.205 | abc |

**TABLE S 9.** Beta diversity and its partitioning according to the Sorensen Baselga family based index.

| **Component** | **Value** | **%** |
| --- | --- | --- |
| BDtotal | 0.43 | 43.04 |
| Repl | 0.43 | 42.65 |
| Nes | 0.00 | 0.40 |
| Repl/BDtotal | 0.99 | 99.08 |
| Nes/BDtotal | 0.01 | 0.92 |

**TABLE S 10.** List of genera differentially expressed between high and low land use intensity in forest ecosystems.

| **LUI** | **Phylum** | **Class** | **Order** | **Family** | **Genus** | **Guild** | **baseMean** | **log2FC** | **lfcSE** | **stat** | **p** | **p adj.** |
| --- | --- | --- | --- | --- | --- | --- | --- | --- | --- | --- | --- | --- |
| High | *Basidiomycota* | *Agaricomycetes* | *Agaricales* | *Amanitaceae* | *Amanita* | symbiotroph | 9.55 | 29.93 | 5.01 | 5.98 | 0 | 0 |
|  | *Basidiomycota* | *Agaricomycetes* | *Agaricales* | *Amanitaceae* | *Amanita* | symbiotroph | 5.64 | 29.87 | 4.88 | 6.13 | 0 | 0 |
|  | *Ascomycota* | *Archaeorhizomycetes* | *Archaeorhizomycetales* | *Archaeorhizomycetaceae* | *Archaeorhizomyces* | saprotroph | 2.89 | 29.78 | 4.04 | 7.37 | 0 | 0 |
|  | *Basidiomycota* | *Agaricomycetes* | *Russulales* | *Russulaceae* | *Russula* | symbiotroph | 38.32 | 28.15 | 4.96 | 5.68 | 0 | 0 |
|  | *Basidiomycota* | *Agaricomycetes* | *Thelephorales* | *Thelephoraceae* | *Thelephora* | symbiotroph | 29.03 | 27.31 | 4.15 | 6.58 | 0 | 0 |
|  | *Basidiomycota* | *Agaricomycetes* | *Atheliales* | *Pilodermataceae* | *Piloderma* | symbiotroph | 20.83 | 27.25 | 4.14 | 6.59 | 0 | 0 |
|  | *Basidiomycota* | *Agaricomycetes* | *Agaricales* | *Hygrophoraceae* | *Hygrophorus* | symbiotroph | 15.47 | 26.87 | 3.9 | 6.9 | 0 | 0 |
|  | *Ascomycota* | *Leotiomycetes* | *Thelebolales* | *Pseudeurotiaceae* | *Pseudeurotium* | saprotroph | 9.57 | 26.08 | 3.94 | 6.62 | 0 | 0 |
|  | *Ascomycota* | *Pezizomycetes* | *Pezizales* | *Pyronemataceae* | *Rhodoscypha* | symbiotroph | 8.65 | 25.98 | 2.77 | 9.36 | 0 | 0 |
|  | *Ascomycota* | *Leotiomycetes* | *Helotiales* | *Hyaloscyphaceae* | *Lachnum* | saprotroph | 5.43 | 25.46 | 3.47 | 7.34 | 0 | 0 |
|  | *Basidiomycota* | *Agaricomycetes* | *Thelephorales* | *Thelephoraceae* | *unclassified* | unknown | 4.29 | 25.1 | 3.85 | 6.53 | 0 | 0 |
|  | *Basidiomycota* | *Agaricomycetes* | *Sebacinales* | *Sebacinaceae* | *Sebacina* | symbiotroph | 5.25 | 24.99 | 4.49 | 5.56 | 0 | 0 |
|  | *Ascomycota* | *Eurotiomycetes* | *Chaetothyriales* | *Herpotrichiellaceae* | *unclassified* | unknown | 3.86 | 24.91 | 3.96 | 6.29 | 0 | 0 |
|  | *Ascomycota* | *Leotiomycetes* | *Helotiales* | *Hyaloscyphaceae* | *Lachnellula* | saprotroph | 19.57 | 24.72 | 1.44 | 17.17 | 0 | 0 |
|  | *Basidiomycota* | *Agaricomycetes* | *Agaricales* | *Hygrophoraceae* | *Cuphophyllus* | saprotroph | 508.86 | 24.66 | 2.12 | 11.66 | 0 | 0 |
|  | *Basidiomycota* | *Agaricomycetes* | *Sebacinales* | *Sebacinaceae* | *Sebacina* | symbiotroph | 2.91 | 24.58 | 5.01 | 4.91 | 0 | 0 |
|  | *Ascomycota* | *Saccharomycetes* | *Saccharomycetales* | *unclassified* | *unclassified* | unknown | 3.01 | 24.18 | 3.29 | 7.36 | 0 | 0 |
|  | *Glomeromycota* | *Glomeromycetes* | *Glomerales* | *Glomeraceae* | *Rhizoglomus* | symbiotroph | 32.16 | 23.77 | 1.79 | 13.3 | 0 | 0 |
|  | *Basidiomycota* | *Agaricomycetes* | *Agaricales* | *Clavariaceae* | *Hodophilus* | saprotroph | 101.03 | 23.72 | 2.63 | 9.03 | 0 | 0 |
|  | *Basidiomycota* | *Agaricomycetes* | *Agaricales* | *Clavariaceae* | *Hodophilus* | saprotroph | 11.62 | 23.48 | 3.81 | 6.17 | 0 | 0 |
|  | *Basidiomycota* | *Agaricomycetes* | *Agaricales* | *Hygrophoraceae* | *Cuphophyllus* | saprotroph | 113.49 | 23.46 | 2.95 | 7.96 | 0 | 0 |
|  | *Ascomycota* | *Sordariomycetes* | *Hypocreales* | *Hypocreaceae* | *Trichoderma* | pathotroph | 12.43 | 22.99 | 2.17 | 10.59 | 0 | 0 |
|  | *Basidiomycota* | *Agaricomycetes* | *Agaricales* | *Clavariaceae* | *Clavaria* | saprotroph | 16.19 | 22.38 | 3.07 | 7.28 | 0 | 0 |
|  | *Ascomycota* | *Eurotiomycetes* | *Onygenales* | *unclassified* | *unclassified* | unknown | 59.99 | 22.06 | 2.52 | 8.75 | 0 | 0 |
|  | *Ascomycota* | *Sordariomycetes* | *Sordariales* | *Chaetomiaceae* | *unclassified* | unknown | 22.97 | 22.03 | 1.72 | 12.78 | 0 | 0 |
|  | *Glomeromycota* | *Glomeromycetes* | *Glomerales* | *Glomeraceae* | *Glomus* | symbiotroph | 8.78 | 22.03 | 2.29 | 9.61 | 0 | 0 |
|  | *Ascomycota* | *Sordariomycetes* | *unclassified* | *unclassified* | *unclassified* | unknown | 32.19 | 21.93 | 1.28 | 17.07 | 0 | 0 |
|  | *Ascomycota* | *Sordariomycetes* | *Hypocreales* | *Incertae sedis* | *Trichothecium* | pathotroph | 8.92 | 21.91 | 1.92 | 11.43 | 0 | 0 |
|  | *Basidiomycota* | *Ustilaginomycetes* | *Ustilaginales* | *Ustilaginaceae* | *Ustilago* | pathotroph | 75.58 | 21.69 | 1.28 | 16.9 | 0 | 0 |
|  | *Basidiomycota* | *Agaricomycetes* | *Agaricales* | *Clavariaceae* | *Hodophilus* | saprotroph | 24.27 | 21.62 | 3.04 | 7.11 | 0 | 0 |
|  | *Ascomycota* | *Saccharomycetes* | *Saccharomycetales* | *Debaryomycetaceae* | *Schwanniomyces* | saprotroph | 268.1 | 21.38 | 0.9 | 23.85 | 0 | 0 |
|  | *Ascomycota* | *Leotiomycetes* | *Helotiales* | *Hyaloscyphaceae* | *Lachnellula* | saprotroph | 24.59 | 21.22 | 2.21 | 9.59 | 0 | 0 |
|  | *Mortierellomycota* | *Mortierellomycetes* | *Mortierellales* | *Mortierellaceae* | *Mortierella* | saprotroph | 18.8 | 21.21 | 1.46 | 14.5 | 0 | 0 |
|  | *Basidiomycota* | *Agaricomycetes* | *Agaricales* | *Hygrophoraceae* | *Hygrocybe* | saprotroph | 21.21 | 21.14 | 2.85 | 7.43 | 0 | 0 |
|  | *Chytridiomycota* | *Spizellomycetes* | *Spizellomycetales* | *unclassified* | *unclassified* | unknown | 18.46 | 21.02 | 1.61 | 13.04 | 0 | 0 |
|  | *Ascomycota* | *Sordariomycetes* | *Microascales* | *Microascaceae* | *Microascus* | saprotroph | 16.11 | 20.79 | 1.97 | 10.54 | 0 | 0 |
|  | *unclassified* | *unclassified* | *unclassified* | *unclassified* | *unclassified* | unknown | 30 | 20.74 | 1.73 | 12.01 | 0 | 0 |
|  | *Ascomycota* | *Dothideomycetes* | *Pleosporales* | *Didymosphaeriaceae* | *Paraphaeosphaeria* | saprotroph | 38.16 | 20.72 | 1.95 | 10.62 | 0 | 0 |
|  | *Basidiomycota* | *Tremellomycetes* | *Filobasidiales* | *Piskurozymaceae* | *Solicoccozyma* | saprotroph | 19.25 | 20.51 | 1.87 | 10.96 | 0 | 0 |
|  | *Basidiomycota* | *Agaricomycetes* | *unclassified* | *unclassified* | *unclassified* | unknown | 9.25 | 20.48 | 2.87 | 7.13 | 0 | 0 |
|  | *Chytridiomycota* | *Rhizophydiomycetes* | *Rhizophydiales* | *unclassified* | *unclassified* | unknown | 44.6 | 20.41 | 1.35 | 15.12 | 0 | 0 |
|  | *Glomeromycota* | *Glomeromycetes* | *Glomerales* | *Glomeraceae* | *Glomus* | symbiotroph | 6.01 | 20.39 | 2.65 | 7.7 | 0 | 0 |
|  | *Basidiomycota* | *Agaricomycetes* | *Agaricales* | *unclassified* | *unclassified* | unknown | 8.23 | 20.39 | 2.62 | 7.79 | 0 | 0 |
|  | *Ascomycota* | *Dothideomycetes* | *Pleosporales* | *Didymellaceae* | *Neoascochyta* | pathotroph | 62.91 | 20.31 | 0.92 | 22.11 | 0 | 0 |
|  | *Mortierellomycota* | *Mortierellomycetes* | *Mortierellales* | *Mortierellaceae* | *Linnemannia* | unknown | 231.13 | 20.17 | 1.1 | 18.41 | 0 | 0 |
|  | *Ascomycota* | *Leotiomycetes* | *Helotiales* | *unclassified* | *unclassified* | unknown | 7.14 | 20.1 | 2.34 | 8.61 | 0 | 0 |
| Low | *Basidiomycota* | *Agaricomycetes* | *Agaricales* | *Amanitaceae* | *Amanita* | symbiotroph | 3.5 | -20.28 | 5.01 | -4.05 | 0 | 0.001 |
|  | *Basidiomycota* | *Microbotryomycetes* | *Sporidiobolales* | *Sporidiobolaceae* | *unclassified* | unknown | 6.82 | -20.32 | 2.07 | -9.84 | 0 | 0 |
|  | *Mortierellomycota* | *Mortierellomycetes* | *Mortierellales* | *Mortierellaceae* | *Linnemannia* | unknown | 17.34 | -20.52 | 1.76 | -11.66 | 0 | 0 |
|  | *Ascomycota* | *Eurotiomycetes* | *Eurotiales* | *Elaphomycetaceae* | *Elaphomyces* | symbiotroph | 6.48 | -22.85 | 5.01 | -4.56 | 0 | 0 |
|  | *Ascomycota* | *Sordariomycetes* | *Myrmecridiales* | *Incertae sedis* | *Atractospora* | saprotroph | 3.81 | -23.29 | 3.23 | -7.22 | 0 | 0 |
|  | *Basidiomycota* | *Agaricomycetes* | *Agaricales* | *Inocybaceae* | *Inocybe* | symbiotroph | 4.28 | -23.63 | 5.01 | -4.72 | 0 | 0 |
|  | *Basidiomycota* | *Agaricomycetes* | *Agaricales* | *Inocybaceae* | *unclassified* | unknown | 5.9 | -23.81 | 5.01 | -4.76 | 0 | 0 |
|  | *Basidiomycota* | *Agaricomycetes* | *Agaricales* | *Clavariaceae* | *Ramariopsis* | saprotroph | 7.67 | -24.62 | 3.89 | -6.33 | 0 | 0 |
|  | *Basidiomycota* | *Agaricomycetes* | *Russulales* | *Russulaceae* | *Russula* | symbiotroph | 92.4 | -25.31 | 4.08 | -6.2 | 0 | 0 |

**TABLE S 11.** List of genera differentially abundant between high and low land use intensity (LUI) in grassland ecosystem.

| **LUI** | **Phylum** | **Class** | **Order** | **Family** | **Genus** | **Guild** | **baseM** | **log2FC** | **lfcSE** | **stat** | **p** | **padj** |
| --- | --- | --- | --- | --- | --- | --- | --- | --- | --- | --- | --- | --- |
| High | *Ascomycota* | *Sordariomycetes* | *Microascales* | *Microascaceae* | *Acaulium* | pathotroph | 8.694 | 35.868 | 2.519 | 14.238 | 0 | 0 |
|  | *Basidiomycota* | *Tremellomycetes* | *Trichosporonales* | *Trichosporonaceae* | *Apiotrichum* | saprotroph | 37.473 | 35.561 | 2.631 | 13.516 | 0 | 0 |
|  | *Ascomycota* | *Sordariomycetes* | *Hypocreales* | *Clavicipitaceae* | *Metapochonia* | pathotroph | 3.938 | 33.748 | 4.416 | 7.642 | 0 | 0 |
|  | *Basidiomycota* | *Microbotryomycetes* | *Leucosporidiales* | *Leucosporidiaceae* | *Leucosporidium* | saprotroph | 3.456 | 32.977 | 2.676 | 12.322 | 0 | 0 |
|  | *Basidiomycota* | *Agaricomycetes* | *Agaricales* | *Tricholomataceae* | *Dermoloma* | saprotroph | 5.359 | 30.207 | 5.008 | 6.032 | 0 | 0 |
|  | *Basidiomycota* | *Agaricomycetes* | *Agaricales* | *Clavariaceae* | *Ramariopsis* | saprotroph | 17.449 | 23.601 | 2.871 | 8.222 | 0 | 0 |
|  | *Ascomycota* | *Leotiomycetes* | *Helotiales* | *Myxotrichaceae* | *Oidiodendron* | saprotroph | 51.1 | 21 | 1.764 | 11.905 | 0 | 0 |
|  | *Ascomycota* | *Sordariomycetes* | *Hypocreales* | *Hypocreaceae* | *Trichoderma* | pathotroph | 6.304 | 20.89 | 2.919 | 7.156 | 0 | 0 |
|  | *Mortierellomycota* | *Mortierellomycetes* | *Mortierellales* | *Mortierellaceae* | *Mortierella* | saprotroph | 26.027 | 20.208 | 1.359 | 14.867 | 0 | 0 |
| Low | *Basidiomycota* | *Agaricomycetes* | *Agaricales* | *Mycenaceae* | *Mycena* | saprotroph | 21.265 | -20.211 | 1.631 | -12.394 | 0 | 0 |
|  | *Ascomycota* | *Leotiomycetes* | *Thelebolales* | *Pseudeurotiaceae* | *Pseudeurotium* | saprotroph | 9.571 | -20.8 | 3.943 | -5.275 | 0 | 0 |
|  | *Basidiomycota* | *Agaricomycetes* | *Agaricales* | *Mycenaceae* | *Mycena* | saprotroph | 51.046 | -20.869 | 1.449 | -14.401 | 0 | 0 |
|  | *Basidiomycota* | *Agaricomycetes* | *Thelephorales* | *Thelephoraceae* | *Tomentella* | symbiotroph | 17.891 | -21.08 | 2.049 | -10.29 | 0 | 0 |
|  | *Rozellomycota* | *unclassified* | *unclassified* | *unclassified* | *unclassified* | unknown | 11.883 | -21.273 | 2.099 | -10.133 | 0 | 0 |
|  | *Mortierellomycota* | *Mortierellomycetes* | *Mortierellales* | *Mortierellaceae* | *unclassified* | unknown | 29.945 | -22.117 | 1.915 | -11.549 | 0 | 0 |
|  | *Basidiomycota* | *Agaricomycetes* | *Sebacinales* | *Sebacinaceae* | *Sebacina* | symbiotroph | 11.81 | -22.231 | 3.191 | -6.968 | 0 | 0 |
|  | *Basidiomycota* | *Agaricomycetes* | *Agaricales* | *Clavariaceae* | *unclassified* | unknown | 5.5 | -24.42 | 3.007 | -8.122 | 0 | 0 |
|  | *Ascomycota* | *Leotiomycetes* | *Thelebolales* | *Pseudeurotiaceae* | *Gymnostellatospora* | saprotroph | 34.277 | -24.834 | 1.299 | -19.116 | 0 | 0 |
|  | *Ascomycota* | *Leotiomycetes* | *Rhytismatales* | *Calloriaceae* | *Polyphilus* | saprotroph | 18.103 | -26.187 | 1.823 | -14.361 | 0 | 0 |
|  | *Ascomycota* | *Dothideomycetes* | *Pleosporales* | *Dictyosporiaceae* | *unclassified* | unknown | 6.841 | -28.338 | 4.591 | -6.172 | 0 | 0 |
|  | *Ascomycota* | *Sordariomycetes* | *Hypocreales* | *Hypocreaceae* | *Hypomyces* | pathotroph | 26.91 | -30.552 | 3.248 | -9.407 | 0 | 0 |
|  | *Ascomycota* | *Geoglossomycetes* | *Geoglossales* | *Geoglossaceae* | *Trichoglossum* | saprotroph | 3.373 | -30.834 | 3.765 | -8.189 | 0 | 0 |
|  | *Ascomycota* | *Dothideomycetes* | *Pleosporales* | *Phaeosphaeriaceae* | *Paraphoma* | pathotroph | 2.764 | -31.252 | 3.581 | -8.728 | 0 | 0 |
|  | *Basidiomycota* | *Agaricomycetes* | *Agaricales* | *Clavariaceae* | *Ramariopsis* | saprotroph | 14.952 | -31.27 | 3.296 | -9.486 | 0 | 0 |
|  | *Ascomycota* | *Leotiomycetes* | *Thelebolales* | *Pseudeurotiaceae* | *Geomyces* | saprotroph | 14.488 | -32.529 | 3.585 | -9.075 | 0 | 0 |
|  | *Basidiomycota* | *Agaricomycetes* | *Agaricales* | *Clavariaceae* | *Clavaria* | saprotroph | 4.414 | -35.158 | 3.476 | -10.116 | 0 | 0 |
|  | *Ascomycota* | *Sordariomycetes* | *Hypocreales* | *unclassified* | *unclassified* | unknown | 11.158 | -35.612 | 3.234 | -11.012 | 0 | 0 |
|  | *Basidiomycota* | *Agaricomycetes* | *unclassified* | *unclassified* | *unclassified* | unknown | 5.232 | -36.493 | 3.936 | -9.273 | 0 | 0 |
|  | *Ascomycota* | *Sordariomycetes* | *Coniochaetales* | *Coniochaetaceae* | *Coniochaeta* | saprotroph | 26.306 | -39.415 | 3.864 | -10.201 | 0 | 0 |
|  | *Basidiomycota* | *Agaricomycetes* | *unclassified* | *unclassified* | *unclassified* | unknown | 28.846 | -41.645 | 3.734 | -11.152 | 0 | 0 |

**TABLE S 12.** List of indicator species in forest and grassland under different levels of land use intensity resulting from the INDVAL test.

| **Ecosystem** | **LUI** | **Phylum** | **Class** | **Order** | **Family** | **Genus** | **Species** | **Guild** | **Secondary lifestyle** | **R^2^** | **p.adj** |
| --- | --- | --- | --- | --- | --- | --- | --- | --- | --- | --- | --- |
| Forest | High | *Ascomycota* | *Saccharomycetes* | *Saccharomycetales* | *unclassified* | *unclassified* | *unclassified* | unknown | unknown | 0.61 | 0.0109 |
|  |  | *Ascomycota* | *Leotiomycetes* | *Helotiales* | *Myxotrichaceae* | *Oidiodendron* | *Oidiodendron chlamydosporicum* | saprotroph | root endophyte | 0.58 | 0.0109 |
|  |  | *Ascomycota* | *Sordariomycetes* | *Hypocreales* | *Hypocreaceae* | *Trichoderma* | *Trichoderma minutisporum* | pathotroph | foliar endophyte | 0.55 | 0.0109 |
|  |  | *Ascomycota* | *Leotiomycetes* | *Helotiales* | *Myxotrichaceae* | *Oidiodendron* | *Oidiodendron chlamydosporicum* | saprotroph | root endophyte | 0.57 | 0.0109 |
|  |  | *Ascomycota* | *Eurotiomycetes* | *Eurotiales* | *Trichocomaceae* | *Sagenomella* | *unclassified* | saprotroph | unknown | 0.55 | 0.0109 |
|  |  | *Ascomycota* | *Leotiomycetes* | *Helotiales* | *Myxotrichaceae* | *Oidiodendron* | *Oidiodendron majus* | saprotroph | root endophyte | 0.60 | 0.0109 |
|  |  | *Ascomycota* | *Leotiomycetes* | *Helotiales* | *Dermateaceae* | *Cryptosporiopsis* | *unclassified* | saprotroph | unknown | 0.63 | 0.0109 |
|  | Low | *Ascomycota* | *Leotiomycetes* | *Thelebolales* | *Pseudeurotiaceae* | *Pseudogymnoascus* | *unclassified* | saprotroph | unknown | 0.56 | 0.0109 |
|  |  | *Ascomycota* | *Sordariomycetes* | *Sordariales* | *Chaetomiaceae* | *unclassified* | *unclassified* | unknown | unknown | 0.59 | 0.0109 |
|  |  | *Ascomycota* | *Pezizomycetes* | *Pezizales* | *Pyronemataceae* | *Leucoscypha* | *unclassified* | saprotroph | unknown | 0.63 | 0.0109 |
|  |  | *Ascomycota* | *Leotiomycetes* | *Helotiales* | *Myxotrichaceae* | *unclassified* | *unclassified* | unknown | unknown | 0.58 | 0.0109 |
|  |  | *Ascomycota* | *Leotiomycetes* | *Helotiales* | *Hyaloscyphaceae* | *unclassified* | *unclassified* | unknown | unknown | 0.65 | 0.0109 |
|  |  | *Ascomycota* | *Pezizomycetes* | *Pezizales* | *Tarzettaceae* | *Tarzetta* | *unclassified* | symbiotroph | unknown | 0.59 | 0.0109 |
|  |  | *Ascomycota* | *Dothideomycetes* | *Pleosporales* | *Lentitheciaceae* | *unclassified* | *unclassified* | unknown | unknown | 0.57 | 0.0109 |
|  |  | *Ascomycota* | *Sordariomycetes* | *Chaetosphaeriales* | *Chaetosphaeriaceae* | *Chloridium* | *Chloridium chloroconium* | saprotroph | wood saprotroph | 0.56 | 0.0109 |
|  |  | *Ascomycota* | *Pezizomycetes* | *Pezizales* | *Pezizaceae* | *Luteoamylascus* | *unclassified* | symbiotroph | unknown | 0.56 | 0.0109 |
|  |  | *Ascomycota* | *Sordariomycetes* | *Chaetosphaeriales* | *Chaetosphaeriaceae* | *unclassified* | *unclassified* | unknown | unknown | 0.55 | 0.0109 |
|  |  | *Ascomycota* | *Dothideomycetes* | *Pleosporales* | *Nigrogranaceae* | *Nigrograna* | *unclassified* | pathotroph | animal decomposer | 0.58 | 0.0109 |
|  |  | *Basidiomycota* | *Tremellomycetes* | *Filobasidiales* | *Piskurozymaceae* | *Solicoccozyma* | *Solicoccozyma terricola* | saprotroph | epiphyte | 0.56 | 0.0109 |
|  |  | *Basidiomycota* | *Agaricomycetes* | *Agaricales* | *Inocybaceae* | *Inosperma* | *Inosperma rimosoides* | unknown | unknown | 0.60 | 0.0109 |
|  |  | *Basidiomycota* | *Agaricomycetes* | *Agaricales* | *Inocybaceae* | *Inosperma* | *unclassified* | unknown | unknown | 0.55 | 0.0109 |
|  |  | *Basidiomycota* | *Agaricomycetes* | *Russulales* | *Russulaceae* | *Lactarius* | *Lactarius subdulcis* | symbiotroph | unknown | 0.56 | 0.0109 |
|  |  | *Basidiomycota* | *Tremellomycetes* | *Tremellales* | *Syzygosporaceae* | *Syzygospora* | *unclassified* | pathotroph | unknown | 0.62 | 0.0109 |
|  |  | *Basidiomycota* | *Agaricomycetes* | *Thelephorales* | *Thelephoraceae* | *Thelephora* | *unclassified* | symbiotroph | unknown | 0.56 | 0.0109 |
|  |  | *Basidiomycota* | *Microbotryomycetes* | *Leucosporidiales* | *Leucosporidiaceae* | *Leucosporidium* | *unclassified* | saprotroph | unknown | 0.56 | 0.0109 |
|  |  | *Mortierellomycota* | *Mortierellomycetes* | *Mortierellales* | *Mortierellaceae* | *Mortierella* | *unclassified* | saprotroph | root-associated | 0.57 | 0.0109 |
|  | Medium | *Basidiomycota* | *Microbotryomycetes* | *Microbotryales* | *unclassified* | *unclassified* | *unclassified* | unknown | unknown | 0.55 | 0.0109 |
|  |  | *Basidiomycota* | *Agaricomycetes* | *Phallales* | *Phallaceae* | *Phallus* | *Phallus impudicus* | saprotroph | unknown | 0.64 | 0.0109 |
| Grassland | High | *Ascomycota* | *Dothideomycetes* | *Pleosporales* | *Sporormiaceae* | *Preussia* | *Preussia flanaganii* | saprotroph | unknown | 0.61 | 0.0109 |
|  |  | *Ascomycota* | *Sordariomycetes* | *Hypocreales* | *Nectriaceae* | *Fusarium* | *Fusarium oxysporum* | pathotroph | litter saprotroph | 0.56 | 0.0109 |
|  |  | *Ascomycota* | *Sordariomycetes* | *Coniochaetales* | *Coniochaetaceae* | *unclassified* | *unclassified* | unknown | unknown | 0.70 | 0.0109 |
|  |  | *Ascomycota* | *Sordariomycetes* | *Hypocreales* | *Bionectriaceae* | *Clonostachys* | *Clonostachys krabiensis* | saprotroph | plant pathogen | 0.65 | 0.0109 |
|  |  | *Ascomycota* | *Sordariomycetes* | *Hypocreales* | *Nectriaceae* | *Fusarium* | *Fusarium graminearum* | pathotroph | litter saprotroph | 0.58 | 0.0109 |
|  |  | *Ascomycota* | *Saccharomycetes* | *Saccharomycetales* | *Debaryomycetaceae* | *Schwanniomyces* | *unclassified* | saprotroph | unknown | 0.60 | 0.0109 |
|  |  | *Ascomycota* | *Dothideomycetes* | *Capnodiales* | *Cladosporiaceae* | *Cladosporium* | *unclassified* | saprotroph | plant pathogen | 0.69 | 0.0109 |
|  |  | *Ascomycota* | *Sordariomycetes* | *Hypocreales* | *Nectriaceae* | *Fusarium* | *Fusarium equiseti* | pathotroph | litter saprotroph | 0.60 | 0.0109 |
|  |  | *Ascomycota* | *Sordariomycetes* | *Hypocreales* | *Nectriaceae* | *Fusicolla* | *Fusicolla aquaeductuum* | pathotroph | fungal decomposer | 0.67 | 0.0109 |
|  |  | *Ascomycota* | *Leotiomycetes* | *Helotiales* | *unclassified* | *unclassified* | *unclassified* | unknown | unknown | 0.65 | 0.0109 |
|  |  | *Ascomycota* | *Sordariomycetes* | *Glomerellales* | *Plectosphaerellaceae* | *Plectosphaerella* | *Plectosphaerella niemeijerarum* | pathotroph | litter saprotroph | 0.60 | 0.0109 |
|  |  | *Ascomycota* | *Dothideomycetes* | *Pleosporales* | *Didymellaceae* | *Neoascochyta* | *Neoascochyta desmazieri* | pathotroph | litter saprotroph | 0.59 | 0.0109 |
|  |  | *Basidiomycota* | *Tremellomycetes* | *Filobasidiales* | *Piskurozymaceae* | *Solicoccozyma* | *Solicoccozyma aeria* | saprotroph | epiphyte | 0.63 | 0.0109 |
|  |  | *Basidiomycota* | *Tremellomycetes* | *Trichosporonales* | *Trichosporonaceae* | *Apiotrichum* | *Apiotrichum dulcitum* | saprotroph | unknown | 0.59 | 0.0109 |
|  |  | *Mortierellomycota* | *Mortierellomycetes* | *Mortierellales* | *Mortierellaceae* | *Linnemannia* | *Linnemannia gamsii* | unknown | unknown | 0.65 | 0.0109 |
|  |  | *Mortierellomycota* | *Mortierellomycetes* | *Mortierellales* | *Mortierellaceae* | *unclassified* | *unclassified* | unknown | unknown | 0.64 | 0.0109 |
|  | Low | *Ascomycota* | *Sordariomycetes* | *Coniochaetales* | *Coniochaetaceae* | *Coniochaeta* | *Coniochaeta verticillata* | saprotroph | foliar endophyte | 0.60 | 0.0109 |
|  |  | *Ascomycota* | *Leotiomycetes* | *Helotiales* | *Incertae sedis* | *Leohumicola* | *Leohumicola verrucosa* | saprotroph | ericoid mycorrhizal | 0.60 | 0.0109 |
|  |  | *Ascomycota* | *Dothideomycetes* | *Pleosporales* | *Cucurbitariaceae* | *Pyrenochaetopsis* | *Pyrenochaetopsis leptospora* | saprotroph | litter saprotroph | 0.61 | 0.0109 |
|  |  | *Ascomycota* | *Sordariomycetes* | *Hypocreales* | *Incertae sedis* | *Acremonium* | *Acremonium persicinum* | saprotroph | foliar endophyte | 0.65 | 0.0109 |
|  |  | *Ascomycota* | *Sordariomycetes* | *Hypocreales* | *Nectriaceae* | *Dactylonectria* | *Dactylonectria macrodidyma* | pathotroph | unknown | 0.61 | 0.0109 |
|  |  | *Ascomycota* | *Sordariomycetes* | *Hypocreales* | *Clavicipitaceae* | *Collarina* | *unclassified* | saprotroph | unknown | 0.58 | 0.0109 |
|  |  | *Ascomycota* | *Eurotiomycetes* | *Chaetothyriales* | *Herpotrichiellaceae* | *unclassified* | *unclassified* | unknown | unknown | 0.59 | 0.0109 |
|  |  | *Mortierellomycota* | *Mortierellomycetes* | *Mortierellales* | *Mortierellaceae* | *Podila* | *Podila minutissima* | unknown | unknown | 0.56 | 0.0109 |
|  | Medium | *Ascomycota* | *Sordariomycetes* | *Glomerellales* | *Plectosphaerellaceae* | *Gibellulopsis* | *Gibellulopsis fusca* | pathotroph | litter saprotroph | 0.58 | 0.0109 |

**TABLE S 13.** Type III Analysis of Variance (ANOVA) permutation test of distance-based redundancy analysis (db-RDA) for the environmental terms considered.

|  | Df | SumOfSqs | F | Pr(>F) |
| --- | --- | --- | --- | --- |
| Type | 1 | 3.095 | 13.170 | 0.001 |
| Medium Sand | 1 | 0.486 | 2.069 | 0.011 |
| IC | 1 | 1.199 | 5.102 | 0.001 |
| pH | 1 | 2.202 | 9.370 | 0.001 |
| CN | 1 | 2.028 | 8.629 | 0.001 |
| TN | 1 | 0.934 | 3.973 | 0.001 |
| Elevation | 1 | 1.236 | 5.258 | 0.001 |
| TC | 1 | 0.949 | 4.040 | 0.001 |
| ind | 2 | 0.932 | 1.983 | 0.004 |
| Coarse Silt | 1 | 0.623 | 2.651 | 0.003 |
| OP | 1 | 0.675 | 2.874 | 0.004 |
| bio13 | 1 | 0.474 | 2.015 | 0.014 |
| Fine Sand | 1 | 0.431 | 1.832 | 0.025 |
| Fine Silt | 1 | 0.464 | 1.975 | 0.012 |
| bio16 | 1 | 0.451 | 1.918 | 0.018 |
| bio12 | 1 | 0.576 | 2.449 | 0.005 |
| bio18 | 1 | 0.510 | 2.172 | 0.011 |
| TS | 1 | 0.404 | 1.719 | 0.039 |
| CS | 1 | 0.381 | 1.620 | 0.046 |
| bio6 | 1 | 0.346 | 1.474 | 0.068 |
| bio19 | 1 | 0.459 | 1.955 | 0.011 |
| bio10 | 1 | 0.398 | 1.692 | 0.031 |
| Coarse Sand | 1 | 0.316 | 1.343 | 0.108 |
| Residual | 275 | 64.62168 |  |  |

**TABLE S 14.** Type III Analysis of Variance (ANOVA) of the distance based redundancy analysis (dbRDA) with selected environmental terms in forest.

|  | Df | SumOfSqs | F | Pr(>F) |
| --- | --- | --- | --- | --- |
| Medium Sand | 1 | 0.463 | 1.981 | 0.009 |
| pH | 1 | 1.379 | 5.905 | 0.001 |
| CN | 1 | 1.077 | 4.610 | 0.001 |
| Elevation | 1 | 0.638 | 2.733 | 0.002 |
| ind | 2 | 0.822 | 1.760 | 0.007 |
| Fine Sand | 1 | 0.576 | 2.466 | 0.003 |
| Coarse Silt | 1 | 0.455 | 1.948 | 0.017 |
| OP | 1 | 0.453 | 1.939 | 0.02 |
| Fine Silt | 1 | 0.444 | 1.901 | 0.016 |
| TS | 1 | 0.465 | 1.993 | 0.009 |
| TC | 1 | 0.660 | 2.825 | 0.001 |
| TN | 1 | 0.629 | 2.694 | 0.004 |
| Residual | 136 | 31.763 |  |  |

**TABLE S 15.** Type III Analysis of Variance (ANOVA) of the distance based redundancy analysis (dbRDA) with selected environmental terms in grassland

|  | **Df** | **SumOfSqs** | **F** | **Pr(>F)** |
| --- | --- | --- | --- | --- |
| IC | 1 | 0.701 | 3.560 | 0.001 |
| pH | 1 | 1.603 | 8.135 | 0.001 |
| Elevation | 1 | 0.807 | 4.096 | 0.001 |
| TN | 1 | 0.360 | 1.826 | 0.016 |
| Medium Sand | 1 | 0.512 | 2.600 | 0.002 |
| CN | 1 | 0.417 | 2.116 | 0.004 |
| OP | 1 | 0.583 | 2.961 | 0.002 |
| ind | 2 | 0.728 | 1.847 | 0.004 |
| bio13 | 1 | 0.381 | 1.934 | 0.009 |
| TS | 1 | 0.389 | 1.973 | 0.01 |
| Fine Sand | 1 | 0.355 | 1.800 | 0.016 |
| Coarse Silt | 1 | 0.295 | 1.497 | 0.062 |
| OC | 1 | 0.283 | 1.437 | 0.078 |
| Residual | 135 | 26.59461 |  |  |
